# Exploring molecular evolution of Rubisco in C_3_ and CAM Orchidaceae and Bromeliaceae

**DOI:** 10.1186/s12862-019-1551-8

**Published:** 2020-01-22

**Authors:** Carmen Hermida-Carrera, Mario A. Fares, Marcel Font-Carrascosa, Maxim V. Kapralov, Marcus A. Koch, Arnau Mir, Arántzazu Molins, Miquel Ribas-Carbó, Jairo Rocha, Jeroni Galmés

**Affiliations:** 1 Research Group on Plant Biology under Mediterranean Conditions, Universitat de les Illes Balears-INAGEA, Ctra. Valldemossa km. 7.5, 07122 Palma, Illes Balears Spain; 2 Integrative and Systems Biology Group, Department of Abiotic Stress, Instituto de Biología Molecular y Celular de Plantas (CSIC–UPV), 46022 Valencia, Spain; 3 Department of Genetics, Trinity College Dublin, University of Dublin, Dublin 2, Ireland; 4 School of Natural and Environmental Sciences, Newcastle University, Newcastle upon Tyne, NE1 7RU United Kingdom; 5 Department of Biodiversity and Plant Systematics, Centre for Organismal Studies (COS) Heidelberg, Heidelberg University, Im Neuenheimer Feld 345, 9120 Heidelberg, Germany; 6 Computational Biology and Bioinformatics Research Group, Department of Mathematics and Computer Science, Universitat de les Illes Balears, 07122 Palma, Illes Balears Spain

**Keywords:** C_3_, CAM, Carboxylation, Catalytic rate, Coevolution, Decision tree, Positive selection, Rubisco

## Abstract

**Background:**

The CO_2_-concentrating mechanism associated to Crassulacean acid metabolism (CAM) alters the catalytic context for Rubisco by increasing CO_2_ availability and provides an advantage in particular ecological conditions. We hypothesized about the existence of molecular changes linked to these particular adaptations in CAM Rubisco. We investigated molecular evolution of the Rubisco large (L-) subunit in 78 orchids and 144 bromeliads with C_3_ and CAM photosynthetic pathways. The sequence analyses were complemented with measurements of Rubisco kinetics in some species with contrasting photosynthetic mechanism and differing in the L-subunit sequence.

**Results:**

We identified potential positively selected sites and residues with signatures of co-adaptation. The implementation of a decision tree model related Rubisco specific variable sites to the leaf carbon isotopic composition of the species. Differences in the Rubisco catalytic traits found among C_3_ orchids and between strong CAM and C_3_ bromeliads suggested Rubisco had evolved in response to differing CO_2_ concentration.

**Conclusions:**

The results revealed that the variability in the Rubisco L-subunit sequence in orchids and bromeliads is composed of coevolving sites under potential positive adaptive signal. The sequence variability was related to δ^13^C in orchids and bromeliads, however it could not be linked to the variability found in the kinetic properties of the studied species.

## Background

Crassulacean acid metabolism (CAM) is one of the three mechanisms found in vascular plants for the assimilation of atmospheric CO_2_. The CAM pathway is characterized by the temporal separation of carbon fixation: CO_2_ is initially fixed by phosphoenolpyruvate carboxylase at night [1–3]. The resulting organic acids are stored in the vacuole and, during the day, decarboxylation of these compounds provides CO_2_ at high concentrations for assimilation by Rubisco [1]. This mechanism makes it possible for CAM plants to close their stomata during the day when the evaporative demand is higher, so the water cost of CO_2_ gain is significantly reduced in CAM plants [2, 3]. This fact, along with other anatomical features that minimize water loss, increases the water use efficiency (WUE) of CAM plants several fold compared to C_3_ and C_4_ plants [4, 5]. The selective advantage of high WUE likely accounts for the extensive diversification and speciation among CAM plants, particularly in water-limited habitats [2, 6]. Indeed, CAM has been reported for 343 genera in 34 families and ca. 7% of all vascular plant species are estimated to exhibit CAM [7–9].

The CO_2_ is considered the central driving force for the earliest evolution of CAM [10, 11]. Actually, it is thought that CAM photosynthesis appeared as the result of adaptive selection related to the decline in the atmospheric CO_2_ concentration and progressive aridification, in a similar manner to the Miocene expansion of grasses with C_4_ mechanism [12–14]. In essence, CAM constitutes a CO_2_-concentrating mechanism originated through daytime malate remobilization from the vacuole and its decarboxylation with regeneration of CO_2_. This mechanism leads to CO_2_ partial pressure ranging between 0.08 and 2.50% in the leaf air spaces during CAM phase III, i.e., when Rubisco is active [15]. With up to 60-fold increase of CO_2_ level in the photosynthesizing organs as compared to the atmospheric CO_2_ partial pressure, this is the strongest known increase of internal CO_2_ partial pressures of CO_2_-concentration mechanisms [16]. This striking increase in CO_2_ concentration might directly impact the functioning of the enzymes in the Calvin cycle, in particular the C_3_ carboxylating enzyme Rubisco, by substrate-saturating its carboxylase activity.

Rubisco has evolved to optimize catalysis according to the availability of CO_2_ in the vicinity of its catalytic sites [17–19]. In principle, optimization of Rubisco to the prevailing environment has to inevitably deal with the trade-off between Rubisco affinity for CO_2_ (1/K_c_) and the enzyme’s turnover rate [17, 18, 20, 21]. Hence, C_3_ species with low CO_2_ concentration at the site of carboxylation, such as those from dry and hot environments, tend to present Rubiscos with higher affinity for CO_2_, albeit with lower maximum rate of carboxylation (*k*_cat_^c^) [19, 22]. On the contrary, in C_4_ plants, with 6–10-fold increase of CO_2_ concentration in the bundle-sheath cells compared to the atmosphere, Rubisco has specialized towards increased *k*_cat_^c^ [23–27].

In the recent years, signatures of positive selection acting on particular amino acid residues of Rubisco have been found using phylogenetic analysis of different taxonomic groups, confirming variation trends in the evolution of the Rubisco kinetics to changing intracellular concentrations of CO_2_ in C_3_ and C_4_ plants [22, 28–34]. In contrast to C_3_ and C_4_ species, the molecular evolution of Rubisco in CAM plants has been poorly investigated. While Kapralov and Filatov (2007) [28] included representatives of CAM pathway in their study, investigation of selection associated with CAM was not among their research objectives.

The exploration of the natural variation of Rubisco catalytic traits by means of molecular and biochemical approaches is far from being complete and is considered a promising way 1) to increase our understanding on how the environmental conditions shape Rubisco evolutionary fine-tuning, and 2) to find Rubisco variants with increased efficiency to use in existing engineering programs aiming at improving Rubisco performance [35, 36]. Previous results, suggesting that high selection pressure on Rubisco has particularly occurred in species from extreme environments and/or possessing innovative adaptations such as carbon concentrating mechanisms [19, 27, 30, 31, 34, 37], make CAM plants a prime subject for understanding mechanisms of evolution in Rubisco.

We undertook the comparative analysis of Rubisco evolution in closely related species from the Orchidaceae and Bromeliaceae families possessing C_3_ and CAM pathways. These two Neotropical plant families represent an outstanding example of adaptive radiation in plants with a striking ecological versatility, occupying habitats extremely different in the ecophysiological demands, among which epiphytic life forms predominate [13, 38–40]. Orchids and bromeliads contain approximately half of the total CAM plant species, and evidence of selection for weak and strong models of CAM has been reported for both families [13, 41–44]. Importantly, CAM pathway evolved several times independently within the Orchidaceae and Bromeliaceae families [3, 40, 45, 46], making them an ideal model to compare Rubisco evolution between CAM and C_3_ related species. Our hypothesis was that a large variability in the L-subunit exists in bromeliads and orchids, and that part of this molecular variability was positively selected to improve the catalytic performance of Rubisco according to the specific physiology of CAM and C_3_ species. To test this hypothesis, we characterized the chloroplast *rbcL* gene to explore the variability of the Rubisco L-subunit within these families and to search for specific amino acid replacements associated with CAM. Thereafter, Rubisco catalytic parameters were measured in representative species to infer the biochemical impact of amino acid replacements within the Rubisco L-subunit. Intra-molecular coevolution analysis was also conducted to further understand the importance and correlation of the Rubisco L-subunit sites under selection with the functionality of Rubisco. Finally, a decision tree (DT) model was implemented to find correlations between Rubisco L-subunit amino acid replacements and essential variables of the species, including carbon isotopic discrimination and habitat preference.

## Results

### Variation of leaf traits and their correlation with the photosynthetic mechanism

As for the purposes of the present study, the classification of the photosynthetic mechanism of the studied species and varieties into different CAM levels is required. Several approaches have been used to determine the presence of CAM, including some leaf morphological traits, such as the leaf thickness, the leaf mass area (LMA) or the FW/DW ratio, indicative of succulence, the leaf carbon isotopic composition (δ^13^C), the time-course of leaf gas-exchange and leaf titratable acidity, and the activity of specific enzymes [3, 5, 13, 42–45, 47–49]. The accurate categorization of species into CAM or C_3_ requires the combination of several of these methods. However, in the present study, due to the number of selected species and to the number and variety of analyses, we discriminated the photosynthetic mechanism on the sole basis of δ^13^C values, and then correlated this with leaf morphological traits in species for which the live specimens were available. The same approach has been applied before in orchids [6, 50–52] and bromeliads [13]. We are aware that whole-tissue δ^13^C alone does not provide a precise indication of the contributions of dark and light CO_2_ fixation to total carbon gain. This is why we adopted a conservative strategy, including the group of weak CAM as a ‘buffer’ between C_3_ and strong CAM.

**Fig. 1:**
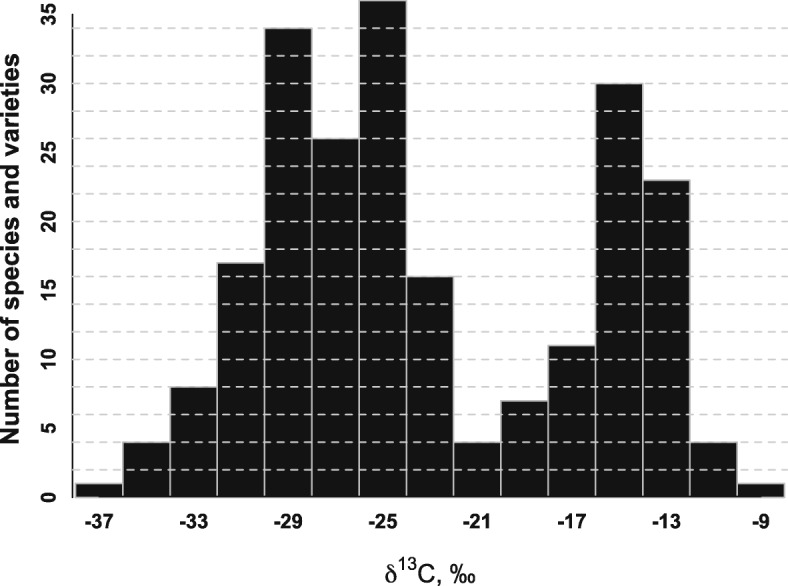
Frequency diagram according to the leaf carbon isotope composition (δ^13^C, ‰) of the 78 orchids and 144 bromeliads studied (see Table 1 and Additional file 1: Table S1)

Among the 78 orchids, the leaf δ^13^C values ranged from − 36.6 to − 12.4 ‰, and from − 33.3 to − 9.4‰ among the 144 bromeliads (Table 1 and Additional file 1: Table S1). The frequency diagram of species and varieties displayed a bimodal distribution with peaks around − 25 and − 15 ‰, in agreement with previous surveys [13, 41, 43, 44, 50, 54] (Fig. 1). Attending to the δ^13^C values, 63 orchids were classified as C_3_ (δ^13^C < − 22.9 ‰) and 15 as CAM (δ^13^C > − 18 ‰). Among the bromeliads, the relative presence of CAM pathway was more evident, with 74 species classified as C_3_, 13 as “weak CAM” and 57 as CAM (Table 1, Additional file 1: Table S1 and Fig. 1).

**Table 1:** List of sequenced Orchidaceae and Bromeliaceae species

Accession No.	Orchids species	δ^13^ C	FW/DW	Leaf thickness	LMA	Habitat preference	Taxon Dataset
MN719136	*Acianthera pubescens* Lindl.	−16.5^c^	7.8 ± 1.1	1.7 ± 0.2	176.7 ± 21.4	Epiphyte	WRHP Taxon No. 2936
MN719137	*Acineta densa* Lindl.	−24.5 ± 0.18	4.7 ± 0.2	0.5 ± 0.1	68.1 ± 3.8	Epiphyte	WRHP Taxon No. 2937
MN719138	*Bulbophyllum lobbii* Lindl.	−26.2 ± 0.89	6.6 ± 0.4	0.5 ± 0.1	68.1 ± 6.5	Epiphyte	WRHP Taxon No. 2938
MN719139	*Elleantus furfuraceus* (Lindl.) Rchb.f	−28.4 ± 0.71	3.1 ± 0.1	0.2 ± 0.1	63.6 ± 5.1	Epiphyte	WRHP Taxon No. 2235
MN719140	*Epidendrum ciliare* L.	−12.4 ^c^	6.4 ± 0.2	1.7 ± 0.1	219.1 ± 11.8	Epiphyte	WRHP Taxon No. 2121
MN719141	*Epidendrum difforme* Jacq.	−25.9 ^c^	13.3 ± 0.9	1.7 ± 0.2	96.5 ± 12.1	Epiphyte	HEID-120487/ HEID-120549
MN719142	*Epidendrum paniculatum* Ruiz & Pav.	−28.4 ^c^	7.0 ± 0.4	0.3 ± 0.1	49.9 ± 3.9	Epiphyte	HEID-120505/ HEID-120651/ HEID-125356
MN719143	*Epidendrum rigidum* Jacq.	−19.8 ± 2.04	6.3 ± 1.2	1.4 ± 0.4	134.6 ± 14.8	Epiphyte	HEID-120499/ HEID-120501
MN719144	*Epidendrum schumannianum* Schltr	−30.4 ^c^	4.7 ± 0.1	0.5 ± 0.1	89.2 ± 6.5	Epiphyte	HEID-125028
MN719145	*Laelia speciosa* (Kunth) Schltr.	−15.4 ± 0.46	6.8 ± 0.5	1.4 ± 0.1	160.5 ± 2.7	Epiphyte	WRHP Taxon No. 1299
MN719146	*Lockhartia amoena* Endres & Rchb.f.	−26.1 ^c^	6.1 ± 0.5	1.7 ± 0.2	150.7 ± 12.4	Epiphyte	WRHP Taxon No. 3006
MN719147	*Lycaste cruenta* (Lindl.) Lindl.	−25.7 ± 0.16	5.7 ± 0.5	0.3 ± 0.1	28.6 ± 2.0	Epiphyte	WRHP Taxon No. 1171
MN719148	*Maxillaria cucullata* Lindl.	−30.7 ± 0.61	4.8 ± 0.3	0.5 ± 0.1	88.8 ± 10.0	Epiphyte, terrestrial, lithophyte	WRHP Taxon N0. 1175
MN719149	*Myoxanthus exasperatus* (Lindl.) Luer.	−27.5 ^c^	5.3 ± 0.1	0.8 ± 0.1	122.6 ± 8.8	Epiphyte	HEID-125088
MN719150	*Oncidium dichromaticum* Rchb.f.	−29.7 ^c^	6.2 ± 0.1	0.5 ± 0.1	70.5 ± 1.9	Epiphyte	HEID-121851
MN719151	*Oncidium lineoligerum* Rchb.f. & Warsz.	−28.3 ^c^	5.9 ± 0.1	0.4 ± 0.1	51.5 ± 1.9	Epiphyte, terrestrial, lithophyte	HEID-121194
MN719152	*Pleurothallis cardiothallis* Rchb.f.	−29 ^c^	9.6 ± 1.2	1.1 ± 0.1	89.1 ± 8.5	Epiphyte	HEID-124116/ HEID-121958/ HEID-121218
MN719153	*Pleurothallis chloroleuca* Lindl.	−28 ^c^	5.4 ± 0.1	0.6 ± 0.1	98.1 ± 5.8	Epiphyte	HEID-125112
MN719154	*Pleurothallis nuda* (Klotsch) Rchb.f.	−25.8 ± 0.44	15.0 ± 2.0	1.5 ± 0.2	65.3 ± 5.3	Epiphyte	HEID-108193
MN719155	*Sobralia macrantha* Lindl.	−26.8 ± 0.44	3.1 ± 0.1	0.6 ± 0.1	83.4 ± 7.2	Epiphyte	WRHP Taxon No. 1017
MN719156	*Stanhopea ecornuta* Lem*.*	−27.1 ^b^	5.4 ± 0.1	0.4 ± 0.1	48.7 ± 1.9	Epiphyte, terrestial, lithophyte	HEID-253783
		−30.2 ^c^ (Average − 28.65)					
Accession No.	Bromeliads species	δ^13^ C	FW/DW	Leaf thickness	LMA	Habitat preference	Taxon Dataset
MN719157	*Billbergia euphemiae* var. *nudiflora* L.B.Smith	−29.6 ^a^	5.6 ± 0.2	0.6 ± 0.1	75.2 ± 5.4	Epiphyte, lithophyte	WRHP Taxon No. 2176
MN719158	*Billbergia euphemiae* E.Morren var. *euphemiae*	−29.6 ^a^	4.4 ± 0.4	0.4 ± 0.1	70.0 ± 1.6	Epiphyte, terrestial, lithophyte	WRHP Taxon No. 2175
MN719159	*Billbergia nutans* H. Wendland ex Regel var. *nutans*	−15.6 ^a^	4.0 ± 0.3	1.1 ± 0.2	204.9 ± 40.2	Epiphyte, terrestial, lithophyte	WRHP Taxon No. 2177
MN719160	*Billbergia vittata* Brongniart	−11.3 ^a^	4.4 ± 0.2	1.2 ± 0.1	200.6 ± 8.5	Epiphyte, lithophyte	WRHP Taxon No. 2178
MN719161	*Cryptanthus fosterianus* L.B.Smith	−13.7 ± 0.19	7.3 ± 0.1	2.4 ± 0.2	138.4 ± 47.2	Terrestrial	WRHP Taxon No. 2179
MN719162	*Cryptanthus glaziovii* Mez	−26.5 ^a^				Lithophyte	WRHP Taxon No. 2180
MN719163	*Cryptanthus scaposus* E.Pereira	−26 ^a^	5.3 ± 0.1	0.8 ± 0.1	75.2 ± 5.3	Terrestrial,	WRHP Taxon No. 2181
MN719164	*Cryptanthus sinuosus* L.B.Smith	−15 ^a^	4.6 ± 0.1	1.3 ± 0.2	174.2 ± 7.2	Terrestrial	WRHP Taxon No. 2182
MN719165	*Cryptanthus warren-loosei* Leme	−17 ^a^	6.6 ± 0.9	0.6 ± 0.1	134.6 ± 14.9	Terrestrial, lithophyte	WRHP Taxon No. 2184
MN719166	*Nidularium fulgens* Lemaire	−14.2 ^a^	3.9 ± 0.1	0.4 ± 0.1	73.4 ± 1.6	Epiphyte, terrestrial, lithophyte	WRHP Taxon No. 1612
MN719167	*Nidularium innocentii* Lemaire var. *innocentii*	−33.3 ^a^	4.4 ± 0.1	0.3 ± 0.1	52.0 ± 6.7	Epiphyte, terrestial	WRHP Taxon No. 2186
MN719168	*Nidularium innocentii* var. *lineatum* (Mez) L.B.Smith	−33.3 ^a^	5.5 ± 0.2	0.4 ± 0.1	49.3 ± 3.0	Epiphyte, terrestial	WRHP Taxon No. 2185
MN719169	*Nidularium innocentii* var. *wittmackianum* (Harms) L.B.Smith	−33.3 ^a^	3.6 ± 0.1	0.3 ± 0.1	54.0 ± 2.2	Epiphyte, terrestial	WRHP Taxon No. 2187
MN719170	*Nidularium purpureum* Beer var. *purpureum*	−13.2 ^a^	3.3 ± 0.1	0.4 ± 0.1	90.0 ± 5.5	Lithophyte,	WRHP Taxon No. 2189
MN719171	*Nidularium regelioides* Ule	−15.9 ^a^	4.2 ± 0.2	0.3 ± 0.1	61.6 ± 5.0	Epiphyte	WRHP Taxon No. 2190
MN719172	*Puya ferruginea* (Ruiz & Pavón) L.B.Smith	−23.2 ^a^	7.7 ± 0.9	1.1 ± 0.1	95.0 ± 3.4	Terrestrial, lithophyte	WRHP Taxon No.2191
MN719173	*Puya humilis* Mez	−23.2 ± 0.20	2.7 ± 0.3	1.2 ± 0.1	363.8 ± 17.5	Terrestrial, lithophyte	WRHP Taxon No. 2198
MN719174	*Puya sanctae-crucis* (Baker) L.B.Smith	−25.9 ^a^	6.4 ± 0.4	2.6 ± 0.1	273.4 ± 15.9	Terrestrial	WRHP Taxon No. 2200
MN719175	*Puya stenothyrsa* (Baker) Mez	−14.8 ^a^	4.9 ± 0.3	3.5 ± 0.3	457.3 ± 17.7	Terrestrial, lithophyte	WRHP Taxon No. 2201
MN719176	*Puya venusta* Philippi	−17.3 ^a^	5.5 ± 0.3	1.3 ± 0.1	175.3 ± 12.7	,Lithophyte	WRHP Taxon No. 2202
MN719177	*Ronnbergia brasiliensis* E.Pereira & Penna	−15.8 ^a^	3.5 ± 0.1	0.4 ± 0.1	90.2 ± 2.6	Epiphyte	WRHP Taxon No. 2203

List of sequenced Orchidaceae and Bromeliaceae species, leaf carbon isotope composition (δ^13^C, ‰), the ratio of leaf fresh mass to dry mass (FW/DW), the leaf thickness (mm), the leaf mass per area (LMA, g m^− 2^) and the habitat preference according to [53]. Values of FW/DW, thickness and LMA are means ± S.E. δ^13^C values which were taken from bibliography are marked with a superscript letter [40^a^, 41^b^, 43^c^] and δ^13^C values without superscript were measured in the present study. The code numbers from the Heidelberg University Botanic Garden are shown (HEID and WRHP, Werner Raugh Heritage Project). See Additional file 1: Table S1 for the complete list of species

FW/DW ranged between 2.7 (*Puya humilis*) and 15 (*Pleurothalis nuda*), the leaf thickness between 0.2 (*Elleantus furfuraceus* and *Tillandsia biflora*) and 3.8 mm (*Puya laxa*), and the LMA between 28.6 (*Lycaste cruenta*) and 457.3 g m^− 2^ (*Puya stenothyrsa*) (Table 1 and Additional file 1: Table S1). Among orchids and bromeliads, strong CAM plants presented significantly (*p* < 0.05) higher LMA than C_3_ plants (Additional file 2: Table S2). The leaf thickness was also significantly (*p* < 0.05) higher in strong CAM compared to C_3_ plants. Higher LMA and leaf thickness in CAM than C_3_ plants have been previously described in taxonomically diverse groups with CAM species [5, 42, 43, 50]. There were no significant differences in FW/DW between strong CAM and C_3_ plants, in agreement with previous surveys in orchids [50], but in disagreement with other surveys in phylogenetically diverse groups [11].

**Fig. 2:**
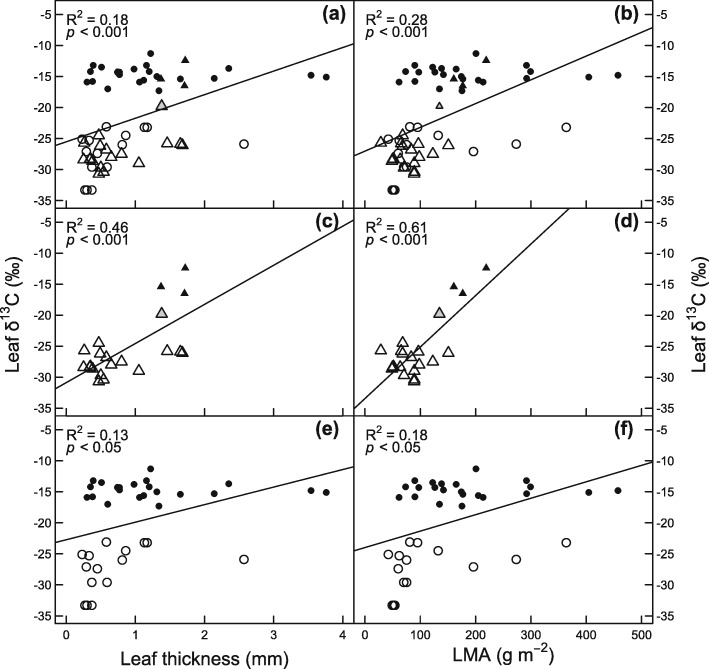
Relationship between the leaf carbon isotope composition (δ^13^C) and the leaf thickness and the leaf mass per area (LMA) for the orchids and bromeliads listed in Table 1 and Additional file 1: Table S1. In (**a**) and (**b**) all orchids and bromeliads are plotted together, in (**c**) and (**d**) only orchids, and in (**e**) and (**f**) only bromeliads. Filled black symbols correspond to strong CAM species of orchids (▲) and bromeliads (●); symbols in grey correspond to weak CAM species of orchids () and bromeliads (); open symbols correspond to C_3_ species of orchids (∆) and bromeliads (○). Values are means (*n* = 4). Regression coefficients between parameters were performed with R [55]

The δ^13^C values tend to be less negative with increasing leaf thickness and LMA when considering bromeliads and orchids together and separately (Fig. 2). As reported in previous findings [41, 50], most species with leaves over 1 mm thick and 100 g m^− 2^ had δ^13^C values less negative than − 18 ‰, indicative of strong CAM. A notable exception was the bromeliad *Puya sanctae-crucis*, with a leaf thickness of 2.6 mm and C_3_-type δ^13^C values (− 25.9 ‰). Exceptions of the correlation between leaf thickness and δ^13^C values have been related to the relative contribution of hydrenchyma to total leaf thickness [10, 43, 50].

### Variability in the Rubisco L-subunit sequence of orchids and bromeliads: analyses of positive selection and intra-molecular coevolution

The L-subunit sequence of bromeliads was 480 amino acids long, while orchid sequences presented diverse length: *Myoxanthus exasperatus, Pleurothallis chloroleuca, P. nuda* and *P. cardiothallis* were 483 amino acids long; *Acianthera pubescens* and *Epidendrum paniculatum* were 479 amino acids long; and the rest of orchid sequences consisted of 481 amino acids. The number of variable amino acid sites was 73 within the orchids and 38 within the bromeliads (Additional file 7: Excel S1 and Additional file 8: Excel S2).

**Fig. 3:**
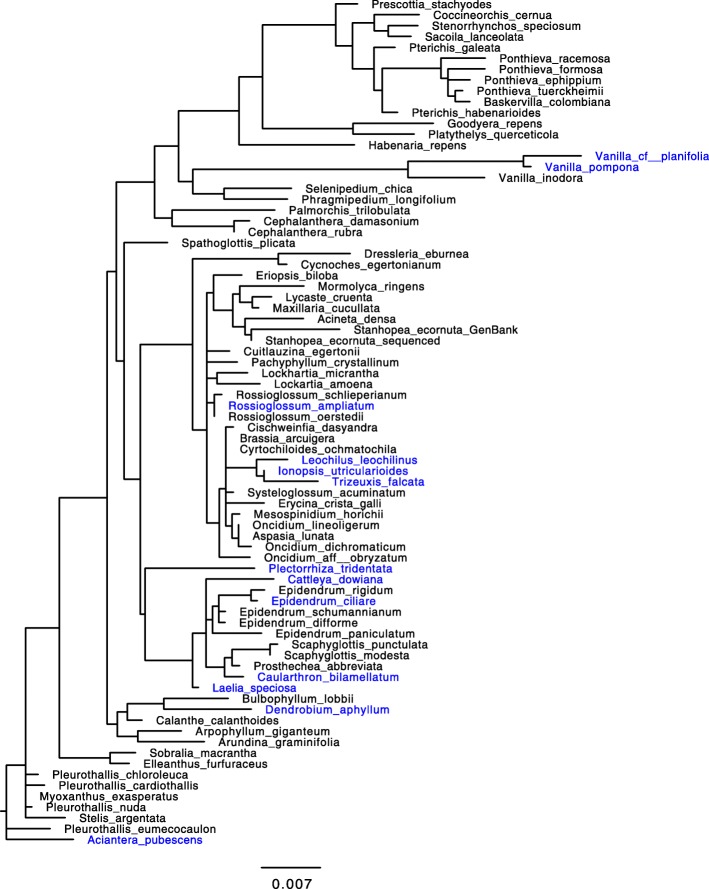
Orchidaceae topology based on *rbcL* sequences (species listed in Table 1 and Additional file 1: Table S1). In blue: CAM species

**Fig. 4:**
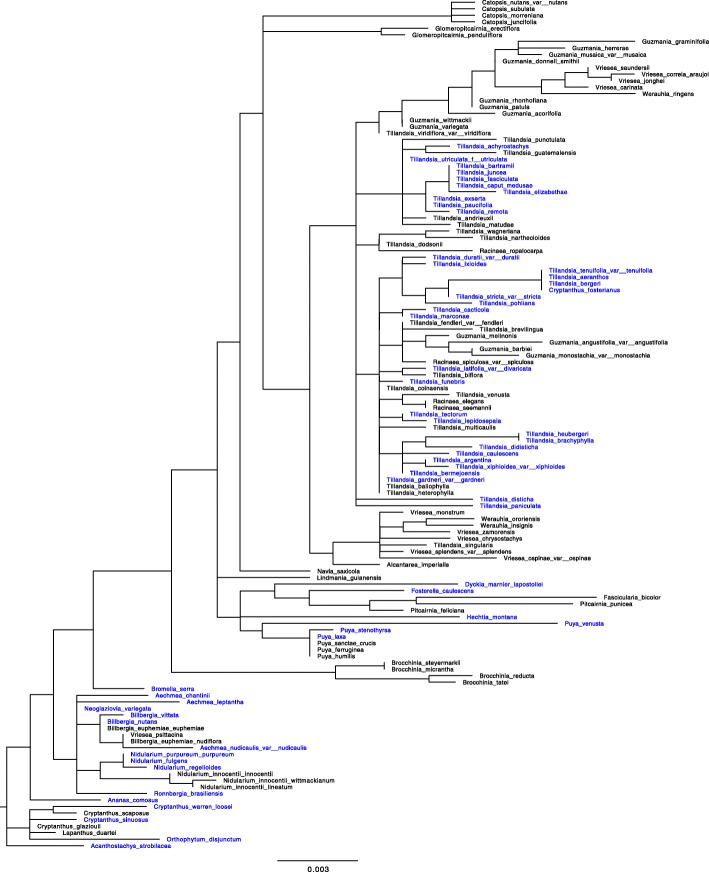
Bromeliaceae topologies based on *rbcL* sequences (species listed in Table 1 and Additional file 1: Table S1). In blue: CAM species

The *rbcL* topologies were constructed for the 78 orchids and 130 bromeliads separately (Figs. 3 and 4). The topology was largely congruent with previously obtained phylogenies [38, 56] and accepted subfamilies divisions.

A total of 13 sites were identified under positive selection within orchids, while within bromeliads signatures of positive selection were identified in 10 sites (Table 2). Common sites under positive selection for both orchids and bromeliads were 142, 225, 251, 449, 468 and 478, while the sites 89, 265, 461, 470, 477, 479 and 481 were exclusive for orchids, and the sites 28, 91, 255 and 270 were found only in bromeliads. However, LRTs (Table 2) indicated that the models assuming positive selection on all branches were not significantly better than the models without positive selection (*p* = 1). For this reason, we will refer to these positively selected sites along the manuscript as candidate sites under positive selection. Moreover, no single codon was identified as evolving under positive selection in branches or clades leading to the CAM species.

**Table 2:** Rubisco L-subunit sites candidate to positive selection

Dataset	N^a^	Site models				M2a vs. M1a test	Site model			M8 vs. M8a test
		M0	M2a				M8			
		*ω*^b^	*p*_2_^c^	*ω*_2_^d^	Selected sites^e^	*p*-value^f^	*p*_1_	*ω*	Selected sites	*p*-value
Orchids	78	0.12	0.89	0.02	89***, 251***, 449**, 461*, 478*, 479**, 481**	1	0.001	999.0	89***, 142*, 225**, 251***, 265*, 449***, 461**, 468**, 470*, 477*, 478*, 479**, 481**	1
Bromeliads	130	0.17	0.91	0.01	28***, 91***, 142***, 225**, 251*, 449**, 468***, 478***	1	0.001	999.0	28***, 91***, 142***, 225***, 251**, 255*, 270*, 449***, 468***, 478***	1

^a^Number of species

^b^*d*N*/d*S ratio averaged across all branches and codons

^c^Proportion of codons in a class under positive selection

^d^*d*N*/d*S ratio in a class under positive selection

^e^Sites marked with *, ** and *** are under positive selection with posterior probability higher than 0.90, 0.95 and 0.99, respectively

^f^*p*-value refers to likelihood ratio tests (LRTs) calculated between nested models of codon evolution M1a-M2a and M8-M8a

Within orchids, 23 amino acidic sites were identified under co-evolution in the L-subunit of Rubisco, distributed in 11 coevolution groups (Additional file 3: Table S3). The residue 475 was the one that appeared as coevolving in more groups, with a total of seven interactions. The residue 466 appeared as coevolving into six groups, and residues 26, 28, 439, 443, 449, 461, 468, 470, 477, 478 and 479 presented four interactions. Other residues with co-evolving interactions were 33, 265, 279, 328, 334, 340, 341, 353, 359 and 447.

Within bromeliads, 20 amino acidic sites were identified under co-evolution, distributed in 2 coevolution groups, being the residues 449 and 478 the ones with two interactions and the rest of co-evolving sites appeared in only one group (Additional file 3: Table S3).

### Decision tree analysis applied to the observed variability in the Rubisco L-subunit

In the orchids dataset, the DT model denoted a link between the external variables (δ^13^C and habitat preference) and the Rubisco L-subunit variable sites 89, 224, 225, 375 (Table 3, Additional file 5: Figure S1). The xerrors calculated for each variable site were 0.96, 0.74, 0.90 and 0.51, respectively. According to the xerror, the sites that were best explained by the external variables were 375 and 224 followed by 225 and 89.

**Table 3:** Variable sites in the Rubisco L-subunit resolved with the DT model for bromeliads and orchids

		Relative importance	
Variable site	xerror	δ^13^C	Habitat preference
Orchids			
89	0.96	85	15
224	0.74	63	37
225	0.90	81	19
375	0.51	65	35
Bromeliads			
91	0.83	100	–
142	0.96	93	7
219	0.93	56	44
225	0.89	100	–
255	0.82	99	1
407	0.66	100	–
464	0.97	100	–
468	0.83	93	7

The xerror correspond to the best DT found for each variable site (x < 1), relative importance (%) of the external variables (δ^13^C and habitat preference) is calculated for each resolved site. Dashes (−) denote not relative importance

In the case of bromeliads, the DT pointed to a link between the variable sites 91, 142, 219, 225, 255, 407, 464 and 468, and the external variables (δ^13^C and habitat preference). The xerrors of 0.83, 0.96, 0.93, 0.89, 0.82, 0.66, 0.97 and 0.83, respectively indicated the site 407 as the best explained by the external variables, followed by 255, 91, 468, 225, 219, 142 and 464 (Table 3, Additional file 6: Figure S2).

For both orchids and bromeliads, the external variable δ^13^C was the one that better correlated with all variable sites (Table 3, Additional files 5: Figure S1 and Additional file 6: Figure S2).

### Rubisco kinetics in orchids and bromeliads: trade-offs and correlation with leaf traits and L-subunit sequence

Among the 5 bromeliads examined, the Rubisco Michaelis-Menten constant affinity for CO_2_ (*K*_c_) varied between 9.6 μM (*T. biflora*) and 27.4 μM (*A. nudicaulis*). Among the 6 orchids examined, *K*_c_ varied between 12.1 μM (*S. macrantha*) and 24.2 μM (*M. cucullata*) (Table 4). The range of variation of the maximum carboxylase rate (*k*_cat_^c^) was similar to that of *K*_c_. Non-significant differences were found in the catalytic carboxylase efficiency (*k*_cat_^c^/*K*_c_) among the selected species. Differences in the relative abundance of Rubisco over leaf total soluble protein ([Rubisco]/[TSP]) were observed among bromeliads (Table 4), with the strong CAM *A. nudicaulis* and *T. bermejoensis* presenting the lowest values.

**Table 4:** Rubisco kinetic parameters at 25 °C and candidate positively selected sites in the Rubisco L-subunit

Species	Photosynthetic type	*K*_c_	*k*_cat_^c^	*k*_cat_^c^*/K*_c_	[Rubisco]/ [TSP]	Candidate L-subunit residuesunder positive selection											
Orchids						89	225	251	265	449	461	468	470	477	478	479	481
* Sobralia macrantha*	C_3_	12.1 ± 1.4^a^	2.5 ± 0.8^ab^	0.21 ± 0.08^a^	8.9 ± 1.5^a^	V	L	I	V	S	I	N	E	Q	L	D	–
* Elleanthus furfuraceus*	C_3_	12.4 ± 0.7^a^	2.8 ± 0.3^ab^	0.22 ± 0.01^a^	7.2 ± 0.7^a^	.	I	.	.	.	.	.	.	K	.	.	–
* Oncidium lineoligerum*	C_3_	14.4 ± 2.3^a^	2.9 ± 0.2^abc^	0.21 ± 0.02^a^	7.2 ± 2.1^a^	A	.	.	.	.	.	D	D	.	E	T	–
* Myoxanthus exasperatus*	C_3_	21.3 ± 1.3^b^	3.7 ± 0.2^bcd^	0.18 ± 0.01^a^	7.6 ± 2.1^a^	.	.	.	.	.	.	.	.	.	.	.	E
* Lockhartia amoena*	C_3_	23.4 ± 0.4^b^	4.3 ± 0.1^cd^	0.18 ± 0.01^a^	6.3 ± 0.3^a^	.	I	.	.	A	.	.	.	.	.	.	–
* Maxillaria cucullata*	C_3_	24.2 ± 4.1^b^	4.4 ± 0.9^d^	0.19 ± 0.01^a^	6.0 ± 0.9^a^	.	.	M	.	.	.	.	.	.	.	.	–
Bromeliads						28	91	142	225	251	449	468	478				
* Tillandsia biflora*	C_3_	9.6 ± 0.7^a^	2.3 ± 0.2^a^	0.25 ± 0.03^a^	18.0 ± 1.1^c^	D	V	P	I	I	C	D	T				
* Tillandsia multicaulis*	C_3_	11.8 ± 0.6^a^	3.2 ± 0.1^a^	0.27 ± 0.02^a^	6.2 ± 0.4^b^	.	.	.	.	.	.	E	A				
* Nidularium innocentii* var. *lineatum*	C_3_	15.5 ± 0.5^a^	3.4 ± 0.6^a^	0.22 ± 0.04^a^	7.2 ± 0.9 ^b^	E	L	.	L	.	S	.	.				
* Tillandsia bermejoensis*	Strong CAM	23.1 ± 3.4^b^	5.7 ± 2.0^a^	0.24 ± 0.06^a^	1.4 ± 0.3^a^	.	.	.	.	.	.	.	.				
* Aechmea nudicaulis* var. *aureo-rosea*	Strong CAM	27.4 ± 2.2^b^	6.4 ± 2.0^a^	0.26 ± 0.09^a^	1.1 ± 0.2^a^	.	.	.	.	.	.	.	.				
* T. aestivum* cv. Cajeme	C_3_	10.3 ± 0.3^a^	1.9 ± 0.1^a^	0.18 ± 0.01^a^	20.9 ± 0.8^d^												

Rubisco kinetics for 6 orchids, 5 bromeliads and *Triticum aestivum*. Rubisco Michaelis-Menten constant for CO_2_ (*K*_c_, μM), maximum rate of carboxylation (*k*_cat_^c^, s^−1^), carboxylase catalytic efficiency (*k*_cat_^c^*/K*_c_, s^−1^ μM^−1^), and Rubisco per leaf total soluble protein ([Rubisco]/[TSP], %). Data are mean ± S.E. (*n* = 3). Different letters denote statistically significant differences among species within orchids and bromeliads through Duncan test (*p* < 0.05). The photosynthetic mechanism is indicated for each species according to Table 1 and Additional file 1: Table S1. The sequence of *T. aestivum* was taken from GenBank (Accesion number KJ592713) for comparison. Residues identical to those of the first sequence are shown as dots

The two studied strong CAM bromeliads (*T. bermejoensis* and *A. nudicaulis*) averaged higher *K*_c_ values (25.3 ± 2.0 μM) compared to the three C_3_ bromeliads (*N. innocentii lineatum*, *T. biflora* and *T. multicaulis*, with 12.3 ± 0.8 μM) (Table 4). Non-significant differences were observed in *k*_cat_^c^/*K*_c_ between strong CAM and C_3_ bromeliads, because *k*_cat_^c^ varied in the same proportion (6.0 ± 1.3 and 3.0 ± 0.2 s^− 1^ for strong CAM and C_3_ bromeliads, respectively).

Apparently, no single amino acid replacement in sites under positive selection (Table 2) or resolved with DT (Table 3) was correlated to the differences observed in *K*_c_ among the studied orchids and bromeliads (Table 4). However, in orchids, the species with low values for *K*_c_ and *k*_cat_^c^, *S. macrantha* and *E. furfuraceus*, presented the potentially positive and predicted replacements 89 V, 468 N, 470E and 478 L, while the species with the highest values for *K*_c_ and *k*_cat_^c^, *L. amoena* and *M. cucullata*, presented 89A, 468D, 470D and 478E.

Correlation coefficients between catalytic parameters, amount of Rubisco, leaf traits and δ^13^C were calculated for all the species using PIC analyses (Table 5). The trade-off between *k*_cat_^c^ and affinity for CO_2_ (1/*K*_c_) was observed in bromeliads and orchids at *P* < 0.001. *K*_c_ and *k*_cat_^c^ correlated significantly with [Rubisco]/[TSP], LMA, leaf thickness and leaf δ^13^C. Finally, [Rubisco]/[TSP] was inversely correlated with LMA, leaf thickness and leaf δ^13^C.

**Table 5:** Phylogenetically Independent Contrasts (PIC)

	*K*_c_	*k*_cat_^c^	*k*_cat_^c^*/K*_c_	[Rubisco]/[TSP]	LMA	Leaf thickness
*k*_cat_^c^	0.989***					
*k*_cat_^c^*/K*_c_	−0.499	−0.392				
[Rubisco]/[TSP]	−0.945**	− 0.971***	0.285			
LMA	0.641*	0.694*	−0.053	− 0.669*		
Leaf thickness	0.886**	0.857**	−0.577*	−0.834**	0.644*	
Leaf δ^13^C	0.863**	0.868**	−0.261	−0.903**	0.453	0.835**

PIC between log transformed Rubisco kinetic parameters, Rubisco per leaf total soluble protein, anatomical and physiological parameters of 11 orchids and bromeliads (see Tables 1, 4 and Additional file 1: Table S1). Rubisco Michaelis-Menten constant for (*K*_c_), maximum rate of carboxylation (*k*_cat_^c^), carboxylation catalytic efficiency (*k*_cat_^c^*/K*_c_), Rubisco per leaf total soluble protein ([Rubisco]/[TSP]), leaf mass area (LMA), leaf thickness and leaf δ^13^C. The table shows the correlations accounting for both orchids and bromeliads together (*n* = 11). Traits that are significantly correlated are marked: *** *p* < 0.001, ** *p* < 0.01, * *p* < 0.05

## Discussion

### Rubisco L-subunit amino acid replacements associated with CAM species

Because water-conserving and CO_2_-concentrating mechanism (CCM) in CAM plants provide an advantage in particular ecological conditions [57, 58], we hypothesized that positive selection of molecular changes promoting such physiological traits may have driven the evolution of CAM Rubisco, in a similar manner of positive adaptive signal associated with C_4_ Rubisco [29–31, 34].

Previous studies reported residues under positive selection in Rubisco L-subunit in different groups of plants [28–30, 33, 34, 59–62] and revealed that amino acid co-evolution is common in Rubisco of land plants [62, 63]. Kapralov and Filatov (2007) [28] reported a number of amino acid sites under positive selection in families sharing C_3_ and CAM species. In the present study, candidate positively selected sites 89, 225, 251 and 265 (in Orchidaceae) and 142, 225, 251 and 255 (in Bromeliaceae) coincided with those reported in their study (Table 2). Sites 142, 449, 461, 468, 470, 477, 478, 479, 481 in orchids, and 28, 91, 270, 449, 468 and 478 in bromeliads are reported in our study but not by Kapralov and Filatov (2007) [28], because of different sample design. Kapralov and Filatov (2007) [28] used different orchids and bromeliads species and fewer of them compared to the present study. Furthermore, all sites under putative positive selection found in this study have been reported in [28] if all phylogenetic groups sampled outside of bromeliads and orchids are taken into account too, confirming widespread convergent evolution within Rubisco among flowering plants [28].

The candidate positive sites 265, 449, 461, 468, 470, 477, 478 and 479 in orchids, and 28, 91, 142, 225, 251, 255, 270, 449 and 468 in bromeliads were identified as coevolving with other amino acid sites (Additional file 3: Table S3). This fact relates positive selection and coevolution within sites located in functionally important interfaces. This is the case of sites 91, 142, 225, 461, 468 and 470, involved in intra-dimer and inter-dimer interactions, interactions with the small subunits and Rubisco Activase, or near to active site (Additional file 4: Table S4).

The candidate positive sites 89 and 225 in orchids, and 91, 142, 225, 255 and 468 in bromeliads were also resolved with a DT (Additional file 4: Table S4). The DT related these sites with the isotopic discrimination, being the species leaf δ^13^C value the most important external variable (Table 3). The apparent discrepancy between the results of branch-site tests of positive selection (no signs of positive selection associated to CAM) and the DT model (amino acid replacements related to δ^13^C) may be attributed to methodological differences. While positive selection analyses were constrained by the binary classification of species into C_3_ or CAM (using labels # in the tree file for the CAM species), the DT model gains from less rigidity as considering numerical values of leaf δ^13^C (all the CAM values of δ^13^C > − 18 ‰ and the C_3_ values < − 22.9 ‰). On the basis of the huge variability in the concentration of CO_2_ at the sites of Rubisco among CAM plants due to the CAM mechanism it seems more appropriate the DT model approach [2, 4]. CAM plants are reported to adjust the expression of different phases in CAM pathway to boost the internal supply of CO_2_ to Rubisco [64, 65]. Recent evidence showed that adaptive forces may act on other regulation points of CAM metabolism, like the enzyme PEPC [66]. It is also important to remark that the δ^13^C values reported in the present study have been obtained from plants grown under different conditions, including greenhouse-grown plants and field data from literature. While this fact was unavoidable to warrant the feasibility of this study, we cannot discard variation in leaf δ^13^C values due to environmental variation.

### Results suggest the existence of differences in the Rubisco kinetics among C_3_ orchids and between C_3_ and strong CAM bromeliads, but the molecular basis of these differences remains to be elucidated

Differences in *K*_c_ and *k*_cat_^c^ at 25 °C were observed among C_3_ orchids but not among C_3_ bromeliads (Table 4). Of the three orchids with higher values of *K*_c_ and *k*_cat_^c^ (*M. exasperatus*, *L. amoena* and *M. cucullata*), *M. exasperatus* and *L. amoena* exhibited large LMA (Table 1 and Additional file 1: Table S1). Higher LMA has been linked to increased mesophyll resistance to CO_2_ transfer and, therefore, low CO_2_ availability at the site of carboxylation [67]. This finding apparently contradicts previous reports suggesting that in C_3_ species low CO_2_ availability promotes Rubisco evolution towards higher affinity for CO_2_ (i.e., low *K*_c_) at the expenses of low *k*_cat_^c^ [19, 33, 37, 68]. The comparison of the particular microclimate where these species evolved may help in understanding the evolutionary causes of this variability among C_3_ orchids. Unfortunately, the lack of success in extracting sufficient active Rubisco in strong CAM orchids precluded the comparison of kinetics between orchids with different photosynthetic mechanism. Future attempts should consider the low concentration of Rubisco present in the leaves of these species, even those with C_3_ mechanism (Table 4).

In bromeliads, the two strong CAM species presented higher *k*_cat_^c^ compared to the C_3_ species (Table 4), although the ratio *k*_cat_^c^/*K*_c_ was similar between the two groups. Higher values of *k*_cat_^c^ at the expense of decreased affinity for CO_2_ (i.e., higher *K*_c_) have been reported in C_4_ plants, and related to the operation of Rubisco at or close to substrate saturation [69]. This finding is in agreement with Lüttge (2011) [16], who reported that the Rubisco specificity for CO_2_/O_2_ (*S*_c/o_) of two CAM species of *Kalanchoë* was at the lower end of the range given for vascular plants. Overall, our results and those by Lüttge (2011) [16] would be indicative of convergent evolution of Rubisco catalysis of C_4_ and CAM plants, in the sense of a retro-evolution under the influence of the internal high CO_2_ concentration. The lower ratio [Rubisco]/[TSP] found in the strong CAM species (Table 4) also mimics the lower content of Rubisco in C_4_ plants [70]. Nevertheless, other studies suggested that CAM Rubiscos retain high CO_2_ affinity (i.e., low Michaelis-Menten constant for CO_2_, *K*_c_) similar to C_3_ plants and lower than C_4_ species [20, 24, 33, 71]. Although the data set available in this study is too small to identify any clear trend, the apparently contradictory results may be attributable to the inherent mechanism of CAM for modulating the relative proportions of Rubisco and PEPC-mediated uptake of atmospheric CO_2_ [64, 71]. Such mechanism determines a wide range of variation in the midday internal CO_2_/O_2_ ratio among different CAM plants [15, 71], and therefore, different degrees of suppression of the oxygenase activity of Rubisco. The apparent variability in Rubisco kinetics associated to CAM could be linked to the plasticity of CAM expression and duration of the different CAM phases and therefore to the different availability of CO_2_ to Rubisco, pointing to the CO_2_ as a driver to Rubisco kinetics evolution [71]. A wide survey on the full Rubisco kinetics including representatives of the different families with CAM species is required to shed light on the evolution of Rubisco kinetics in CAM plants.

The candidate sites under positive selection (Table 2) and resolved with DT (Table 3) in orchids and bromeliads with contrasting Rubisco kinetics did not provide any clear trend on the molecular determinants of L-subunit variability (Table 4). While the present results reveal that there are potential differences in Rubisco traits between phylogenetically related C_3_ and CAM species, more data are needed to confirm this trend and to link kinetic differences to amino acid replacements within the L-subunit. Although not well understood yet, the different expression of *rbcS* genes, encoding for the small subunit (S-subunit) of Rubisco, may allow optimizing the Rubisco performance in response to changing environmental conditions [30, 72–74]. In view of the phenotypic plasticity inherent of the CAM metabolism [4, 64] a role of the S-subunit in the catalysis of Rubisco may not be discarded and should be a matter of future studies. Alternatively, the fact that genes with similar kinetic properties have different amino acid sequences could mean that different lineages used different replacements to lead to the same kinetic changes.

## Conclusion

This study presents an extensive analysis of Rubisco molecular and biochemical characterization in two angiosperm families with C_3_ and CAM photosynthetic pathways. The study includes, for the first time, analyses of closely related C_3_ and CAM species, in particular i) positive selection and coevolution analyses, along with a DT model for variable sites related to physiological and anatomical information, and ii) measurements of Rubisco *k*_cat_^c^ and *K*_c_, that permitted to explore the variability in the Rubisco L-subunit sequences and study their biochemical impact. Signal of positive selection was found in *rbcL* and it could be linked to CAM through the DT. The previously reported trade-off between *K*_c_ and *k*_cat_^c^ was observed in a subset of studied species, with strong CAM bromeliads presenting high *k*_cat_^c^ while C_3_ bromeliads presenting high affinity for CO_2_ (i.e., low *K*_c_). In spite of the differences between C_3_ and CAM bromeliads, the observed variation in the kinetic properties of Rubisco from distinct photosynthetic pathways could not be related to positively selected residues in the Rubisco L-subunit. A deeper inspection of variation in the Rubisco L- and S-subunits and Rubisco biochemical traits across a larger number of families containing C_3_ and CAM species may help to resolve these questions.

## Methods

### Species selection

We selected orchids and bromeliads *rbcL* sequences from GenBank with δ^13^C data available from literature: 123 bromeliads and 58 orchids species (Additional file 1: Table S1). In addition, we included other 42 species for which *rbcL* was sequenced in the present study (Table 1). Therefore, the final list of species under study contained 144 bromeliads and 79 orchids.

### rbcL *amplification and sequencing*

For the 42 sequenced species the genomic DNA was isolated from dry leaves using a DNeasy Plant Mini Kit (Quiagen Ltd., Crawley, UK) in accordance with the manufacturer’s protocol. For *rbcL* amplification, we designed the primers esp2F (5′-AATTCATGAGTTGTAGGGAGGGACTT-3′), B1R (5′-CAATTAGGAGAACAAAGAGGAA-3′), O2F (5′-GAGTAGACCTTGTTGTTGTG-3′) and 1925R (5′-GACACGAGATTCTACGAGA-3′), and used 1494R (5′-GATTGGGCCGAGTTTAATTTAC-3′) [75]. The BioMix Red reaction mix (Bioline Ltd., London, UK) was used to carry out the polymerase chain reaction (PCR) with the following conditions: 1 initial cycle of 95 °C, 2 min; 55 °C, 30 s; 72 °C, 4 min followed by 36 cycles of 93 °C, 30 s; 53 °C, 30 s; 72 °C, 3.5 min. PCR products were visualized on 1% agarose gels, purified using the High Pure PCR Product Purification Kit (Roche, Germany) and sequenced with an ABI 3130 Genetic analyzer and the contings were assembled using BioEdit v7.1.3 software [76]. Novel sequences have been submitted to GenBank (Table 1). Nucleotide sequences were converted into amino acidic sequences with MEGA 5 [77] and then aligned using MAFFT v5 [78].

### Plant material

Among the full dataset of sequences, there were a total of 58 living specimens available at Heidelberg Botanical Garden (Heidelberg University, Germany) (Table 1 and Additional file 1: Table S1). The growth conditions in the glasshouses corresponded approximately to their natural environmental conditions. Natural daylight was supplemented by additional artificial light (photon flux density of 275 μmol m^− 2^ s^− 1^) all over the year. From May until October the glasshouses were partially shaded (approx. 65%). Day-time and night-time minimum temperatures were in the range of 18–20 °C and 14–18 °C, respectively. Relative humidity was kept within the range 70–95%. Plants were watered daily and using a conventional nutrient solution once a week. There were some special cases, e.g., *Puya* was cultivated under dry conditions and full sun light. All ‘grey tillandsia’ were kept outside the glasshouse (shaded as described above) from May to October with daily watering, but they were kept much dryer during the winter season, when these plants do not grow and rest.

Species and varieties were classified according to their habitat preference into epiphyte, terrestrial or lithophyte [53]. A complete documentation is accessible at [79].

### Leaf traits, carbon isotopic composition and photosynthetic mechanism classification

Material for leaf traits and carbon isotopic composition determination consisted in four fully expanded leaves (replicates) in mature stage sampled from different individuals in June 2014.

After the thickness of the leaf lamina was measured between the leaf margin and midrib of the middle portions of leaves using a slide caliper (Vernier Caliper, Series 530, Mitutoyo Europe GmbH), the leaf was detached and the fresh weight (FW) immediately recorded. The leaf area of the same sample was measured after digitalizing the leaf and using the ImageJ software [80]. The dry weight (DW) was obtained after drying the leaves in a ventilated oven at 60 °C until constant weight (typically after 2 days). The leaf mass area (LMA) was calculated as the ratio between the dry weight and the area.

Values for the leaf carbon isotopic composition (δ^13^C) were taken from bibliography (Table 1 and Additional file 1: Table S1), except for *Acineta densa, Bulbophyllum lobbii, Elleantus furfuraceus, Epidendrum rigidum, Laelia speciosa, Lycaste cruenta, Maxillaria cucullata, Pleurothallis nuda, Sobralia macrantha, Cryptanthus fosterianus* and *Puya humilis* for which it was measured. The dried leaves used for the characterization of the leaf traits were ground into powder and subsamples of 2 mg were analyzed. Samples were combusted in an elemental analyzer (Carlo-Erba, Rodano, Italy). The CO_2_ was separated by chromatography and directly injected into a continuous-flow isotope ratio mass spectrometer (Thermo Finnigan Delta Plus, Bremen, Germany). Peach leaf standards (NIST 1547) were run every six samples. The δ^13^C was calculated as: δ^13^C sample (‰) = (((^13^C/^12^C) sample/(^13^C/^12^C) standard) - 1) 1000 [81] and values were referred to a Pee Dee Belemnite standard.

The photosynthetic mechanism of the species was inferred from the δ^13^C values, following previous surveys in orchids and bromeliads [43, 45]. Species with δ^13^ C > − 18 ‰ and < − 22.9 ‰ were classified as CAM and C_3_ respectively. In literature, species with δ^13^ C between − 18 ‰ and − 22.9 ‰ are considered as “weak CAM” (Fig. 1). According to Winter and Holtum (2002) [82], δ^13^C values below − 25 ‰ may indicate that CO_2_ fixation occurs exclusively in the light, while δ^13^C values above − 21.9 ‰ reflect that at least 50% of CO_2_ fixation occurs in the dark.

### *Detection of positive selection in* rbcL

Positive selection acting on the Rubisco L-subunit was analyzed with the PAML package v4.7 [83] and PAMLX [84]. Codeml program [85] was used to calculate the non-synonymous (*d*_N_) and synonymous (*d*_S_) substitution rates across codons and the *d*_N_/*d*_S_ ratio (*ω*). This ratio represents the selective pressures acting on the protein-coding gene with values of *ω* < 1, *ω* = 1, and *ω* > 1 being indicative of purifying selection, neutral evolution and positive selection, respectively.

The tree topologies based on *rbcL* sequences were constructed using maximum-likelihood inference conducted with RAxML version 7.2.6 [86]. It was done without species with δ^13^ C values between − 18.0 and − 22.9 ‰ because species with values around − 20 ‰ might be weak CAM and other may be pure C_3_ with no detectable CAM [41]. Therefore, the tree topologies were finally constructed with 78 orchids and 130 bromeliads (Figs. 3 and 4) and edited with Fig Tree v 1.4.0 [87].

Site models allow the *ω* ratio to vary among codons in the protein [88]. To identify signatures of adaptive evolution we performed two nested maximum likelihood tests: M1a vs. M2a and M8a vs. M8 [89, 90]. The null M1a model assumes purifying selection or nearly neutral evolution without positive selection and allow codons with *ω* < 1 and/or *ω* = 1, but not codons with *ω* > 1. The M2a model allows for codons under positive selection (*ω* > 1). Model M8a assumes a discrete beta distri- bution for *ω*, which is constrained between 0 and 1 including a class with *ω* = 1. Model M8 allows the same distribution as M8a with an extra class of codons under positive selection with *ω* > 1. Posterior probabilities for site classes were calculated with Bayes Empirical Bayes (BEB) [90].

Branch-site models allow *ω* to vary both among sites in the protein and across branches on the tree with the aim to detect positive selection affecting a few sites along particular branches. The branch-site A model was applied for branches leading to CAM species and for clades containing CAM species. The branch types are specified using labels in the tree file; e.g. if the dataset has CAM branch types, they are labelled using #. Species with δ^13^ C > − 18 ‰ were classified as CAM. The A1-A LRT compared the null model A1 with the nested model A. Both the A1 and A models allow ω ratios to vary among sites [83, 91]. The A1 model allows 0 < *ω* < 1 and *ω* = 1 for all branches and also two additional classes of codons with fixed *ω* = 1 along pre-specified branches, while restricted as 0 < *ω* < 1 and *ω* = 1 on background branches. The alternative A model allows 0 < *ω* < 1 and *ω* = 1 for all branches and also two alternative classes of codons under positive selection with ω > 1 along pre-specified branches, while restricted as 0 < *ω* < 1 and *ω* = 1 on background branches.

We performed three LRTs to compare the nested site models M1a-M2a, M8-M8a and branch-site models A-A1. LRTs involves the comparison of the log-likelihood values of the simple and the complex nested models and twice their difference follows a chi-square distribution with the degrees of freedom (df) being the difference in the number of free parameters between the models. For the comparison of models M1a-M2a, M8a-M8 and A1-A the df was 2, 1 and 1, respectively.

### Analysis of intra-molecular coevolution in the amino acidic sequence of the Rubisco large subunit

Intra-molecular coevolution analysis was performed with the program CAPS [92, 93]. The algorithm implemented in this program identifies co-evolving amino acid site pairs by measuring the correlated evolutionary variation at these sites using time corrected Blosum values. CAPS take into account the time of sequences divergence such that correlated variation that involves radical amino acid substitutions is considered to be more likely at longer evolutionary times following a Poisson model [92, 93]. Accordingly, the transition between two amino acids at each site is corrected by the divergence time of the sequences. Synonymous substitutions per site do not affect the amino acid composition of the protein and are neutrally fixed in the gene, being the number of such substitutions proportional to the time of sequence divergence. In this respect, time since two sequences diverge is estimated as the mean number of substitutions per synonymous site between the two sequences being compared. Correlation of the mean variability is measured using the Pearson coefficient. The significance of the correlation coefficient is estimated by comparing the real correlation coefficients to the distribution of resampled correlation coefficients.

### Decision tree (DT) model

DT model analysis (‘rpart’ package in R v3.1.1 [55]) was used to relate the proportion of amino acid presence in all variable sites of the L-subunit of Rubisco to species-specific traits (δ^13^C and habitat preference), denoted as *external variables*, as listed in Table 1 and Additional file 1: Table S1.

For each variable site, the program builds a DT as follows. Based on the external variables (δ^13^C and habitat preference), the species are separated into two groups, in which the variability of that site is as low as possible. The analysis is repeated for each subgroup using again the two external variables. The process continues until the lowest xerror [94] for the entire DT is obtained. In the case of δ^13^C as an external variable the whole range of numerical values were considered, species with δ^13^ C > − 18 ‰ and < − 22.9 ‰, and in the case of habitat as an external variable, three options were possible for each species (epiphyte, lithophyte, terrestrial), so we have given a proportional value (0.34, 0.33, 0.33) for the construction of the DT.

The quality of the DT is categorized by its entropic error (xerror) as a function of the proportion of correct predictions and the complexity of the tree. The lower the xerror, the higher the correlation between the external variable and the variable site. Only DTs with xerror < 1 were selected. The relative importance of an external variable is computed as a function of the reduction of errors that the selected external variable produces on the variable site.

### Rubisco kinetics measurements

For the catalytic characterization of Rubisco, a number of orchid and bromeliad species was selected as representing the different photosynthetic types and reflecting the maximum variability in positively selected residues of the Rubisco L-subunit sequence. The list of species initially selected was: *Acineta densa, Bulbophyllum lobbii, Epidendrum ciliare, Epidendrum difforme, Nidularium fulgens, Nidularium innocentii* var. *innocentii, Nidularium regelioides, Maxillaria cucullata, Oncidium lineoligerum, Lockhartia amoena, Elleanthus furfuraceus, Myoxanthus exasperatus, Sobralia macrantha, Nidularium innocentii lineatum, Tillandsia biflora, Tillandsia multicaulis, Aechmea nudicaulis* var. *aureo-rosea* and *Tillandsia bermejoensis.* Specimens of these species sent from Heidelberg were grown in the glasshouse at the University of the Balearic Islands under similar conditions described for Heidelberg.

Different protein extraction media were tested on these species. These tests determined that the most appropriate protein extraction media were buffers A and B. Extraction buffer A consisted of 100 mM Bicine-NaOH (pH 8.0), 0.1 mM EDTA, PEG4000 (6% w/v), 20 mM DTT, 50 mM 2-mercaptoethanol, 2 mM MgCl_2_, 10 mM NaHCO_3_, 1 mM benzamidine, 1 mM β-aminocaproic acid, 2 μM pepstatin, 10 μM E-64 (Sigma, USA) 10 μM chymostatin, 2 mM phenylmethylsulfonyl fluoride and 25 mg mL^− 1^ PVP. Extraction buffer B consisted of 350 mM HEPES-KOH (pH 8.0), 6% (w/v) PEG4000, 2 mM MgCl_2_, 0.1 mM EDTA, 1 mM benzamidine, 1 mM ε-aminocaproic acid, 10 mM NaHCO_3_. Added into the mortar: 7 μL β-mercaptoethanol, 400 μL DTT (1 M), 4 μL pepstatine, 4 μL E-64, 4 μL chymostatin, 10 μL PMSF, 75 mg PVP and 75 mg PVPP.

Extraction buffer A worked with *M. cucullata, O. lineoligerum, L. amoena, E. furfuraceus, M. exasperatus, S. macrantha, N. innocentii lineatum, T. biflora* and *T. multicaulis.* Leaf soluble protein of *A. nudicaulis* var. *aureo-rosea* and *T. bermejoensis* was successfully extracted using buffer B.

As for the remaining species, *A. densa, B. lobbii, E. ciliare, E. difforme, N. fulgens, N. innocentii* var. *innocentii, N. regelioides,* these two buffers yielded poor soluble protein and low Rubisco activity, and up to other four extraction buffers were tested by varying both the components and their concentration. However, none of these buffers extracted sufficient amount of active Rubisco to characterize the kinetic constants.

Leaf soluble protein was extracted on fully expanded leaves of 3–4 plants per species by grinding 0.40–0.60 g of leaf samples in a mortar with 2 mL of ice-cold extraction buffer. The proportion of leaf total soluble protein that is accounted for by Rubisco ([Rubisco]/[TSP]), the Rubisco Michaelis-Menten constant for CO_2_ (*K*_c_) and the maximum rate of carboxylation (*k*_cat_^c^) were measured at 25 °C in semi-purified extracts following [33]. Rates of Rubisco ^14^CO_2_-fixation using the activated protein extract were measured in 7 mL septum capped scintillation vials in reaction buffer (110 mM Bicine-NaOH pH 8.0, 22 mM MgCl_2_, 0.4 mM RuBP and ~ 100 W-A units of carbonic anhydrase), equilibrated with nitrogen (N_2_). Different concentrations of H^14^CO_3_^−^ (0, 6.7, 26.7, 53.3, 88.9, 122.2 and 155.6 μM for orchids, and 0, 6.7, 26.7, 53.3, 88.9, 122.2, 155.6 and 190 μM for bromeliads; each with a specific radioactivity of 3.7 × 10^10^ Bq mol^− 1^) were prepared in the scintillation vials as described previously [33]. Assays (1.0 mL total volume) were started by injection of activated leaf extract and stopped after 60 s with the addition of 1 M formic acid. The acidified mixtures were dried and the ^14^C products determined via scintillation counting. Concentrations of CO_2_ in solution in equilibrium with H^14^CO_3_^−^ were calculated assuming a pK_a_ for carbonic acid of 6.23.

*Triticum aestivum* cv. Cajeme was grown from seeds at the UIB under full irrigation and frequent fertilization with Hoagland’s solution [95]. Rubisco was extracted from wheat mature leaves using extraction buffer A, and the kinetic parameters measured following the same procedures as with orchids and bromeliads.

### Statistical analyses

Univariate analysis of variance (ANOVA) was used to statistically examine the differences among species and photosynthetic mechanisms for the Rubisco kinetic parameters, [Rubisco]/[TSP] and leaf mass per unit area (LMA). Phylogenetic Independent Contrast (PIC) analysis was performed using R packages APE and GEIGER [96, 97]. Significant differences between means were revealed by Duncan analyses (*p* < 0.05) [98]. Regression coefficients between parameters were performed with R [55].

## Supplementary information

**Additional file 1: Table S1.** List of Orchidaceae and Bromeliaceae species downloaded from GenBank, accession number, leaf carbon isotope composition (δ^13^C, ‰), the ratio of leaf fresh mass to dry mass (FW/DW), the leaf thickness (mm), the leaf mass per area (LMA, g m^− 2^) and the habitat preference according to [53].

**Additional file 2: Table S2.** Mean ± S.E. (*n* = 4) for the leaf mass per area (LMA), the leaf thickness and the leaf fresh to dry weight ratio (FW/DW) of C_3_, weak CAM and strong CAM for orchids and bromeliads. Values for the individual species are shown in Table 1 and Additional file 1: Table S1. Different letters denote statistically significant differences among metabolic types through Duncan test (*p* < 0.05).

**Additional file 3: Table S3.** Coevolving groups of residues detected within the L-subunit of Rubisco within orchids and bromeliads.

**Additional file 4: Table S4.** Integrative view of the Rubisco L-subunit variable sites under positive selection, coevolving and resolved with DT model as a function of external variables (δ^13^C and habitat preference).

**Additional file 5: Figure S1**. Decision trees (DT) resolved for each Rubisco L-subunit variable site as a function of the external variables leaf δ^13^C (‰) and habitat preference for the orchids dataset.

**Additional file 6: Figure S2**. Decision trees (DT) structure resolved for each variable site as a function of the external variables leaf δ^13^C (‰) and habitat preference based on the bromeliads dataset.

**Additional file 7: Excel S1**. Rubisco L-subunit variable sites among orchids. Dots: the same amino acid as the first species in the list. Dash: not available.

**Additional file 8: Excel S2**. Rubisco L-subunit variable sites among bromeliads. Dots: the same amino acid as the first species in the list. Dash: not available.

## Data Availability

The datasets supporting the conclusions of this article are included within the article and its additional files.

## Abbreviations

ANOVA: Analysis of variance
BEB: Bayes Empirical Bayes
CAM: Crassulacean acid metabolism
DT: Decision tree
DTT: Dithiothreitol
DW: Dry weight
EDTA: Ethylenediaminetetraacetic acid
FW: Fresh weight
LMA: Leaf mass area
LRT: Likelihood ratio test
L-subunit: Large subunit
PCR: Polymerase chain reaction
PEG: Polyethylene glycol
PEPC: Phosphoenolpyruvate carboxylase
PIC: Phylogenetic independent contrast
PVP: Polyvinylpyrrolidone
PVPP: Polyvinylpolypyrrolidone
RuBP: Ribulose 1,5-bisphosphate
TSP: Total soluble protein
WUE: Water use efficiency
