# Supplementary material for: Exploring molecular evolution of Rubisco in C3 and CAM Orchidaceae and Bromeliaceae
Source: BMC Evol Biol. 2020 Jan 22;20:11. doi: 10.1186/s12862-019-1551-8 (PMC6977233; doi:10.1186/s12862-019-1551-8)
Supplement: Supplementary file 1 — Additional file 1: Table S1. List of Orchidaceae and Bromeliaceae species downloaded from GenBank, accession number, leaf carbon isotope composition (δ13C, ‰), the ratio of leaf fresh mass to dry mass (FW/DW), the leaf thickness (mm), the leaf mass per area (LMA, g m− 2) and the habitat preference according to [53]. [file 12862_2019_1551_MOESM1_ESM.docx]

**Additional file 1: Table S1.** List of Orchidaceae and Bromeliaceae species downloaded from GenBank, accession number, leaf carbon isotope composition (δ^13^C, ‰), the ratio of leaf fresh mass to dry mass (FW/DW), the leaf thickness (mm), the leaf mass per area (LMA, g m^-2^) and the habitat preference according to [53]. Values of FW/DW, thickness and LMA are means ± S.E..Values of δ^13^ C were taken from [40^a^, 41^b^, 43^c^, 99^d^, 100^e^, 101^f^, 102^g^, 103^h^, 104^i^]. The code numbers from the Heidelberg University Botanic Garden are shown (WRHP, Werner Raugh Heritage Project).

| Accession No. | Orchids species | δ^13^ C | FW/DW | Leaf thickness | LMA | Habitat preference | Taxon Dataset |  |
| --- | --- | --- | --- | --- | --- | --- | --- | --- |
| AF074110 | *Arpophyllum giganteum* Hartw. ex Lindl. | -25.1 ^c^ |  |  |  | Epiphyte | [WRHP Taxon No. 2881](http://scriptorium.cos.uni-heidelberg.de/php/taxon_details.php?tx=2881) |  |
| AF074111 | *Arundina graminifolia* (D. Don) Hochr. | -26.7 ^c^ |  |  |  | Terrestrial | [WRHP Taxon No. 2882](http://scriptorium.cos.uni-heidelberg.de/php/taxon_details.php?tx=2882) |  |
|  |  | -26.5 ^b^ (Average -26.6) |  |  |  |  |  |  |
| FJ534160 | *Aspasia lunata* Lindl. | -31.3 ^b^ |  |  |  | Epiphyte | [WRHP Taxon No. 2883](http://scriptorium.cos.uni-heidelberg.de/php/taxon_details.php?tx=2883) |  |
| AM778157 | *Baskervilla colombiana* Garay. | -34.5 ^c^ |  |  |  | Terrestrial | [WRHP Taxon No. 2884](http://scriptorium.cos.uni-heidelberg.de/php/taxon_details.php?tx=2884) |  |
| FJ534192 | *Brassia arcuigera* Rchb. f. | -28 ^c^ |  |  |  | Epiphyte, terrestrial | [WRHP Taxon No. 2885](http://scriptorium.cos.uni-heidelberg.de/php/taxon_details.php?tx=2885) |  |
|  |  | -25.2 ^b^ (Average -26.6) |  |  |  |  |  |  |
| AF264159 | *Calanthe calanthoides* (A. Rich. & Galeotti) Hamer & Garay. | -32 ^c^ |  |  |  | Epiphyte, terrestrial | [WRHP Taxon No. 2886](http://scriptorium.cos.uni-heidelberg.de/php/taxon_details.php?tx=2886) |  |
| AF074122 | *Cattleya dowiana* Bateman. | -12.7 ^c^ |  |  |  | Epiphyte | [WRHP Taxon No. 2887](http://scriptorium.cos.uni-heidelberg.de/php/taxon_details.php?tx=2887) |  |
|  |  | -16.2 ^b^ (Average -14.45) |  |  |  |  |  |  |
| AF518059 | *Caularthron bilamellatum* (Rchb. f.) R.E. Schult. | -14.3 ^c^ |  |  |  | Epiphyte | [WRHP Taxon No. 2888](http://scriptorium.cos.uni-heidelberg.de/php/taxon_details.php?tx=2888) |  |
| AF074123 | *Cephalanthera damasonium* (Mill.) Druce. | -28.1^b^ |  |  |  | Terrestrial | [WRHP Taxon No. 2889](http://scriptorium.cos.uni-heidelberg.de/php/taxon_details.php?tx=2889) |  |
| JX088502 | *Cephalanthera rubra* (L.) Rich. | -28.6^b^ |  |  |  | Terrestrial | [WRHP Taxon No. 2890](http://scriptorium.cos.uni-heidelberg.de/php/taxon_details.php?tx=2890) |  |
| FJ534248 | *Cischweinfia dasyandra* (Rchb. f.) Dressler & N.H. Williams. | -28 ^c^ |  |  |  | Epiphyte | [WRHP Taxon No. 2891](http://scriptorium.cos.uni-heidelberg.de/php/taxon_details.php?tx=2891) |  |
|  |  | -30.3 ^b^ (Average -29.15) |  |  |  |  |  |  |
| AJ542422 | *Coccineorchis cernua* (Lindl.) garay. | -31 ^c^ |  |  |  | Terrestrial | [WRHP Taxon No. 2892](http://scriptorium.cos.uni-heidelberg.de/php/taxon_details.php?tx=2892) |  |
| FJ534166 | *Cuitlauzina egertonii* (Lindl.) Dressler & N.H. Williams. | -23.2 ^c^ |  |  |  | Epiphyte, terrestrial | [WRHP Taxon No. 2893](http://scriptorium.cos.uni-heidelberg.de/php/taxon_details.php?tx=2893) |  |
| AY368355 | *Cycnoches egertonianum* Bateman. | -31.4 ^c^ |  |  |  | Epiphyte | [WRHP Taxon No. 2894](http://scriptorium.cos.uni-heidelberg.de/php/taxon_details.php?tx=2894) |  |
| FJ534150 | *Cyrtochiloides ochmatochila* (Rchb. f.) N.H. Williams & M.W. Chase. | -24.6 ^c^ |  |  |  | Epiphyte | [WRHP Taxon No. 2895](http://scriptorium.cos.uni-heidelberg.de/php/taxon_details.php?tx=2895) |  |
|  |  | -26.7 ^b^ (Average -25.65) |  |  |  |  |  |  |
| KF177573 | *Dendrobium aphyllum* (Roxb.) C.E.C. Fisch. | -13.9 ^h^ |  |  |  | Epiphyte, lithophyte | [WRHP Taxon No. 2896](http://scriptorium.cos.uni-heidelberg.de/php/taxon_details.php?tx=2896) |  |
| AF074153 | *Dressleria eburnea* (Rolfe) Dodson *.* | -25.3 ^c^ |  |  |  | Epiphyte | [WRHP Taxon No. 2897](http://scriptorium.cos.uni-heidelberg.de/php/taxon_details.php?tx=2897) |  |
| AF074167 | *Eriopsis biloba* Lindl. | -28.5 ^c^ |  |  |  | Epiphyte, terrestrial | [WRHP Taxon No. 2096](http://scriptorium.cos.uni-heidelberg.de/php/taxon_details.php?tx=2096) |  |
| FJ534164 | *Erycina crista-galli* (Rchb. f.) N.H. Williams & M.W. Chase. | -27 ^c^ |  |  |  | Epiphyte, lithophyte | [WRHP Taxon No. 2899](http://scriptorium.cos.uni-heidelberg.de/php/taxon_details.php?tx=2899) |  |
|  |  | -24.1 ^b^ (Average -25.55) |  |  |  |  |  |  |
| FJ571330 | *Goodyera repens* (L.) R. Br. | -36.6 ^i^ |  |  |  | Terrestrial | [WRHP Taxon No. 2900](http://scriptorium.cos.uni-heidelberg.de/php/taxon_details.php?tx=2900) |  |
| AF074177 | *Habenaria repens* Nutt. | -31.1 ^c^ |  |  |  | Terrestrial | [WRHP Taxon No. 2901](http://scriptorium.cos.uni-heidelberg.de/php/taxon_details.php?tx=2901) |  |
| FJ534225 | *Ionopsis utricularioides* (Sw.) Lindl. | -12.6 ^c^ |  |  |  | Epiphyte | [WRHP Taxon No. 1590](http://scriptorium.cos.uni-heidelberg.de/php/taxon_details.php?tx=1590) |  |
|  |  | -12.7 ^b^ (Average -12.65) |  |  |  |  |  |  |
| FJ534149 | *Leochilus leochilinus* (Rchb. f.) M.W. Chase & N.H. Williams. | -13.6 ^c^ |  |  |  | Epiphyte | [WRHP Taxon No. 2902](http://scriptorium.cos.uni-heidelberg.de/php/taxon_details.php?tx=2902) |  |
| FJ534134 | *Lockhartia micrantha* Rchb. f. | -24.9 ^c^ |  |  |  | Epiphyte | [WRHP Taxon No. 2904](http://scriptorium.cos.uni-heidelberg.de/php/taxon_details.php?tx=2904) |  |
|  |  | -24 ^b^ (Average -24.45) |  |  |  |  |  |  |
| FJ534185 | *Mesospinidium horichii* I. Bock. | -29.3 ^c^ |  |  |  | Epiphyte | [WRHP Taxon No. 2905](http://scriptorium.cos.uni-heidelberg.de/php/taxon_details.php?tx=2905) |  |
| AY368363 | *Mormolyca ringens* (Lindl.) Schltr. | -24.1 ^c^ |  |  |  | Epiphyte | [WRHP Taxon No. 2903](http://scriptorium.cos.uni-heidelberg.de/php/taxon_details.php?tx=2903) |  |
| FJ534169 | *Oncidium obryzatum* Rchb. f. & Warsz. | -28.6 ^c^ |  |  |  | Epiphyte | [WRHP Taxon No. 2906](http://scriptorium.cos.uni-heidelberg.de/php/taxon_details.php?tx=2906) |  |
| FJ534233 | *Ornithocephalus dressleri* (Toscano) Toscano & Dressler. | -19 ^c^ |  |  |  | Epiphyte | [WRHP Taxon No.  2916](http://scriptorium.cos.uni-heidelberg.de/php/taxon_details.php?tx=2916) |  |
| FJ534232 | *Pachyphyllum crystallinum* Lindl. | -30.6 ^c^ |  |  |  | Epiphyte, terrestrial, lithophyte | [WRHP Taxon No. 2917](http://scriptorium.cos.uni-heidelberg.de/php/taxon_details.php?tx=2917) |  |
| AF074206 | *Palmorchis trilobulata* L.O. Williams. | -35.1 ^c^ |  |  |  | Terrestrial | [WRHP Taxon No. 2918](http://scriptorium.cos.uni-heidelberg.de/php/taxon_details.php?tx=2918) |  |
| AY918855 | *Phragmipedium longifolium* (Warsz. & Rchb. f.) Rolfe. | -37.1 ^c^ |  |  |  | Terrestrial, lithophyte | [WRHP Taxon No. 2919](http://scriptorium.cos.uni-heidelberg.de/php/taxon_details.php?tx=2919) |  |
|  |  | -29.7 ^b^ (Average -33.4) |  |  |  |  |  |  |
| AF074216 | *Platythelys querceticola* (Lindl.) Garay. | -34.4 ^c^ |  |  |  | Terrestrial | [WRHP Taxon No. 2920](http://scriptorium.cos.uni-heidelberg.de/php/taxon_details.php?tx=2920) |  |
| FN870901 | *Plectorrhiza tridentate* (Lindl.) Dockr. | -15.4 ^h^ |  |  |  | Epiphyte | [WRHP Taxon No. 2921](http://scriptorium.cos.uni-heidelberg.de/php/taxon_details.php?tx=2921) |  |
| AF264174 | *Pleurothallis eumecocaulon* Schltr. | -28 ^c^ |  |  |  | Epiphyte, terrestial | [WRHP Taxon No. 2922](http://scriptorium.cos.uni-heidelberg.de/php/taxon_details.php?tx=2922) |  |
| AM778155 | *Ponthieva ephippium* Rchb. f. | -30.1 ^c^ |  |  |  | Terrestrial | [WRHP Taxon No. 2923](http://scriptorium.cos.uni-heidelberg.de/php/taxon_details.php?tx=2923) |  |
| AM778159 | *Ponthieva formosa* Schltr. | -27.9 ^c^ |  |  |  | Epiphyte | [WRHP Taxon No. 2924](http://scriptorium.cos.uni-heidelberg.de/php/taxon_details.php?tx=2924) |  |
| AJ542417 | *Ponthieva racemosa* (Walter) C. Mohr. | -29.2 ^c^ |  |  |  | Terrestrial | [WRHP Taxon No. 2925](http://scriptorium.cos.uni-heidelberg.de/php/taxon_details.php?tx=2925) |  |
| AM778160 | *Ponthieva tuerckheimii* Schltr. | -34.2 ^c^ |  |  |  | Terrestrial | [WRHP Taxon No. 2926](http://scriptorium.cos.uni-heidelberg.de/php/taxon_details.php?tx=2926) |  |
| AM778139 | *Prescottia stachyodes* (Sw.) Lindl. | -30.5 ^c^ |  |  |  | Terrestrial | [WRHP Taxon No. 2927](http://scriptorium.cos.uni-heidelberg.de/php/taxon_details.php?tx=2927) |  |
| AF518063 | *Prosthechea abbreviata* (Schltr.) W.E. Higgins. | -25 ^c^ |  |  |  | Epiphyte | [WRHP Taxon No. 2928](http://scriptorium.cos.uni-heidelberg.de/php/taxon_details.php?tx=2928) |  |
|  |  | -26.5 ^b^ (Average -25.75) |  |  |  |  |  |  |
| AM778162 | *Pterichis galeata* Lindl. | -28.3 ^c^ |  |  |  | Terrestrial | [WRHP Taxon No. 2935](http://scriptorium.cos.uni-heidelberg.de/php/taxon_details.php?tx=2935) |  |
| AJ542416 | *Pterichis habenarioides* (F. Lehm. & Kraenzl.) Schltr. | -27.9 ^c^ |  |  |  | Terrestrial | [WRHP Taxon No. 2934](http://scriptorium.cos.uni-heidelberg.de/php/taxon_details.php?tx=2934) |  |
| FJ534135 | *Rossioglossum ampliatum* (Lindl.) M.W. Chase & N.H. Williams. | -16 ^c^ |  |  |  | Epiphyte | [WRHP Taxon No. 2933](http://scriptorium.cos.uni-heidelberg.de/php/taxon_details.php?tx=2933) |  |
| FJ534167 | *Rossioglossum oerstedii* (Rchb. f.) M.W. Chase & N.H. Williams. | -32.1 ^c^ |  |  |  | Epiphyte, terrestial | [WRHP Taxon No. 2932](http://scriptorium.cos.uni-heidelberg.de/php/taxon_details.php?tx=2932) |  |
| FJ534136 | *Rossioglossum schlieperianum* Rchb. f. | -26.9 ^c^ |  |  |  | Epiphyte | [WRHP Taxon No. 2931](http://scriptorium.cos.uni-heidelberg.de/php/taxon_details.php?tx=2931) |  |
| AJ542441 | *Sacoila lanceolata* (Aubl.) Garay. | -28.1 ^c^ |  |  |  | Terrestrial | [WRHP Taxon No. 2930](http://scriptorium.cos.uni-heidelberg.de/php/taxon_details.php?tx=2930) |  |
| AF518067 | *Scaphyglottis modesta* (Rchb. f.) Schltr. | -29.7 ^c^ |  |  |  | Epiphyte, lithophyte | [WRHP Taxon No. 2939](http://scriptorium.cos.uni-heidelberg.de/php/taxon_details.php?tx=2939) |  |
| AF518064 | *Scaphyglottis punctulata* (Rchb. f.) C. Schweinf. | -28.5 ^c^ |  |  |  | Epiphyte, terrestial | [WRHP Taxon No. 2929](http://scriptorium.cos.uni-heidelberg.de/php/taxon_details.php?tx=2929) |  |
| AF074227 | *Selenipedium chica* Rchb. f. | -31.8 ^c^ |  |  |  | Terrestrial | [WRHP Taxon No. 2915](http://scriptorium.cos.uni-heidelberg.de/php/taxon_details.php?tx=2915) |  |
| AF264175 | *Spathoglottis plicata* Blume. | -29.9 ^c^ |  |  |  | Terrestrial |  |  |
|  |  | -28.9 ^b^ (Average -29.4) |  |  |  |  |  |  |
| AF074230 | *Stanhopea ecornuta* Lem*.* | -27.1 ^b^ | 5.4 ± 0.1 | 0.4 ± 0.1 | 48.7 ± 1.9 | Epiphyte, terrestrial, lithophyte | HEID-253783 |  |
|  |  | -30.2 ^c^  (Average -28.65) |  |  |  |  |  |  |
| AF518043 | *Stelis argentata* Lindl. | -30.4 ^c^ |  |  |  | Epiphyte | [WRHP Taxon No. 2913](http://scriptorium.cos.uni-heidelberg.de/php/taxon_details.php?tx=2913) |  |
| AJ542420 | *Stenorrhynchos speciosum* (Jacq.) Rich. ex Spreng. | -32.6 ^c^ |  |  |  | Epiphyte, terrestial | [WRHP Taxon No. 2912](http://scriptorium.cos.uni-heidelberg.de/php/taxon_details.php?tx=2912) |  |
| FJ534165 | *Systeloglossum acuminatum* Ames & C. Schweinf. | -30.7 ^c^ |  |  |  | Epiphyte | [WRHP Taxon No. 2911](http://scriptorium.cos.uni-heidelberg.de/php/taxon_details.php?tx=2911) |  |
| FJ534151 | *Trizeuxis falcata* Lindl. | -12.8 ^c^ |  |  |  | Epiphyte | [WRHP Taxon No. 2910](http://scriptorium.cos.uni-heidelberg.de/php/taxon_details.php?tx=2910) |  |
| AF074242 | *Vanilla planifolia* Andrews. | -16.4 ^b^ |  |  |  | Epiphyte | [WRHP Taxon No. 2909](http://scriptorium.cos.uni-heidelberg.de/php/taxon_details.php?tx=2909) |  |
|  |  | -15.3 ^c^ (Average -15.85) |  |  |  |  |  |  |
| AY381136 | *Vanilla inodora* Schiede. | -29 ^c^ |  |  |  | Epiphyte | [WRHP Taxon No. 2908](http://scriptorium.cos.uni-heidelberg.de/php/taxon_details.php?tx=2908) |  |
| FN545555 | *Vanilla pompona* Schiede. | -15.9 ^c^ |  |  |  |  | [WRHP Taxon No. 2907](http://scriptorium.cos.uni-heidelberg.de/php/taxon_details.php?tx=2907) |  |
|  |  | -16.5 ^b^ (Average -16.2) |  |  |  | Epiphyte |  |  |
|  |  |  |  |  |  |  |  |  |
| Accession No. | Bromeliads species | δ13 (‰) | FW/DW | Leaf thickness | LMA | Habitat Preference | Taxon Dataset |  |
| KC123345 | *Acanthostachys strobilacea* (Schult. f.) Link, Klotzsch & Otto. | -16.1^a^ |  |  |  | Epiphyte | [WRHP Taxon No. 2799](http://scriptorium.cos.uni-heidelberg.de/php/taxon_details.php?tx=2799) |  |
| L19978 | *Aechmea chantinii* (Carrière) Baker. | -13.6 ^a^ |  |  |  | Epiphyte | [WRHP Taxon No. 2800](http://scriptorium.cos.uni-heidelberg.de/php/taxon_details.php?tx=2800) |  |
| KC123380 | *Aechmea leptantha* (Harms) Leme & J.A.Siqueira. | -11.8 ^a^ |  |  |  | Epiphyte, terrestial, lithophyte | [WRHP Taxon No. 2818](http://scriptorium.cos.uni-heidelberg.de/php/taxon_details.php?tx=2818) |  |
| AY614390 | *Aechmea nudicaulis* (L.) Griseb. var. *nudicaulis* (L.) Griseb. | -14.7^d^ | 6.5 ± 1.5 | 0.8 ± 0.1 | 141.8 ± 13.9 | Epiphyte, terrestial, lithophyte | [WRHP Taxon No. 1741](http://scriptorium.cos.uni-heidelberg.de/php/taxon_details.php?tx=1741) |  |
|  |  | -14.7 ^a^ |  |  |  |  |  |  |
|  |  | -13.1 ^f^ (Average -14.2) |  |  |  |  |  |  |
| AY614397 | *Alcantarea duarteana* (L.B. Sm.) J.R. Grant. | -21.5 ^a^ |  |  |  | Lithophyte | [WRHP Taxon No. 2819](http://scriptorium.cos.uni-heidelberg.de/php/taxon_details.php?tx=2819) |  |
| AY614398 | *Alcantarea imperialis* (Carrière) Harms. | -24.6 ^a^ |  |  |  | Lithophyte | [WRHP Taxon No. 2820](http://scriptorium.cos.uni-heidelberg.de/php/taxon_details.php?tx=2820) |  |
|  |  | -24.3 ^a^ (Average -24.45) |  |  |  |  |  |  |
| L19977 | *Ananas comosus* (L.) Merr*.* | -15 ^a^ |  |  |  | Terrestrial, lithophyte | [WRHP Taxon No. 2821](http://scriptorium.cos.uni-heidelberg.de/php/taxon_details.php?tx=2821) |  |
|  |  | -13.9 ^f^ |  |  |  |  |  |  |
|  |  | -12.4 ^d^ (Average -13.7) |  |  |  |  |  |  |
| AY614381 | *Brocchinia micrantha* (Baker) Mez. | -25.7 ^a^ |  |  |  | Terrestrial, lithophyte | [WRHP Taxon No. 1737](http://scriptorium.cos.uni-heidelberg.de/php/taxon_details.php?tx=1737) |  |
| AY614384 | *Brocchinia reducta* Baker. | -27.7 ^a^ |  |  |  | Terrestrial | [WRHP Taxon No. 2802](http://scriptorium.cos.uni-heidelberg.de/php/taxon_details.php?tx=2802) |  |
|  |  | -22.6 ^f^ (Average -25.15) |  |  |  |  |  |  |
| AY614382 | *Brocchinia steyermarkii* L.B. Sm. | -24.1 ^a^ |  |  |  | Terrestrial | [WRHP Taxon No. 2803](http://scriptorium.cos.uni-heidelberg.de/php/taxon_details.php?tx=2803) |  |
| AY614383 | *Brocchinia tatei* L.B. Sm. | -27.6 ^a^ |  |  |  | Epiphyte, terrestial, lithophyte | [WRHP Taxon No. 1276](http://scriptorium.cos.uni-heidelberg.de/php/taxon_details.php?tx=1276) |  |
| AY614389 | *Bromelia plumieri* (E. Morren) L.B. Sm. | -20.4 ^d^ |  |  |  | Terrestrial, lithophyte | [WRHP Taxon No. 2822](http://scriptorium.cos.uni-heidelberg.de/php/taxon_details.php?tx=2822) |  |
| KC123350 | *Bromelia serra* Griseb. | -11.7 ^a^ |  |  |  | Terrestrial | [WRHP Taxon No. 2804](http://scriptorium.cos.uni-heidelberg.de/php/taxon_details.php?tx=2804) |  |
| AY614393 | *Catopsis juncifolia* Mez & Wercklé. | -24.3 ^a^ |  |  |  | Epiphyte | [WRHP Taxon No. 1320](http://scriptorium.cos.uni-heidelberg.de/php/taxon_details.php?tx=1320) |  |
| AY614391 | *Catopsis morreniana* Mez. | -24.4 ^a^ |  |  |  | Epiphyte | [WRHP Taxon No. 1765](http://scriptorium.cos.uni-heidelberg.de/php/taxon_details.php?tx=1765) |  |
| AY614392 | *Catopsis nutans* Sw. var. *nutans* (Sw.) Griseb. | -24.9 ^a^ |  |  |  | Epiphyte | [WRHP Taxon No. 1153](http://scriptorium.cos.uni-heidelberg.de/php/taxon_details.php?tx=1153) |  |
| AY614394 | *Catopsis subulata* L.B. Sm. | -29 ^a^ |  |  |  | Epiphyte | [WRHP Taxon No. 1503](http://scriptorium.cos.uni-heidelberg.de/php/taxon_details.php?tx=1503) |  |
| FJ861120 | *Dyckia marnier-lapostollei* L.B. Sm. | -13.8 ^a^ |  |  |  | Terrestrial, lithophyte | [WRHP Taxon No. 1681](http://scriptorium.cos.uni-heidelberg.de/php/taxon_details.php?tx=1681) |  |
| KM360783 | *Fascicularia bicolor* (Ruiz & Pav.) Mez. | -23.8 ^a^ |  |  |  | Epiphyte, lithophyte | [WRHP Taxon No. 2808](http://scriptorium.cos.uni-heidelberg.de/php/taxon_details.php?tx=2808) |  |
| HQ182428 | *Fosterella caulescens* Rauh. | -24.8 ^a^ |  |  |  | Terrestrial | [WRHP Taxon No. 1534](http://scriptorium.cos.uni-heidelberg.de/php/taxon_details.php?tx=1534) |  |
| AY614395 | *Glomeropitcairnia erectiflora* Rauh. | -33.7 ^d^ |  |  |  | Epiphyte | [WRHP Taxon No. 2810](http://scriptorium.cos.uni-heidelberg.de/php/taxon_details.php?tx=2810) |  |
|  |  | -27.5 ^a^(Average -30.6) |  |  |  |  |  |  |
| AY614396 | *Glomeropitcairnia penduliflora* (Griseb.) Mez. | -28.2 ^a^ |  |  |  | Terrestial, epiphyte | [WRHP Taxon No. 2811](http://scriptorium.cos.uni-heidelberg.de/php/taxon_details.php?tx=2811) |  |
| AY614426 | *Guzmania* acorifolia (Griseb.) Mez. | -24.7 ^a^ |  |  |  | Terrestial, epiphyte | [WRHP Taxon No. 2814](http://scriptorium.cos.uni-heidelberg.de/php/taxon_details.php?tx=2814) |  |
| AY614418 | *Guzmania angustifolia* (Baker) Wittm. var. *angustifolia* | -29.6^a^ |  |  |  | Terrestial, epiphyte | [WRHP Taxon No. 2046](http://scriptorium.cos.uni-heidelberg.de/php/taxon_details.php?tx=2046) |  |
| AY614419 | *Guzmania barbiei* Rauh. | -25.7 ^a^ |  |  |  | Terrestrial | [WRHP Taxon No. 2815](http://scriptorium.cos.uni-heidelberg.de/php/taxon_details.php?tx=2815) |  |
| AY614429 | *Guzmania donnellsmithii* Mez ex Donn. Sm. | -30.7 ^a^ |  |  |  | Epiphyte | [WRHP Taxon No. 2816](http://scriptorium.cos.uni-heidelberg.de/php/taxon_details.php?tx=2816) |  |
| AY614423 | *Guzmania graminifolia* (André ex Baker) L.B. Sm. | -29.8 ^a^ |  |  |  | Epiphyte, terrestial, lithophyte | [WRHP Taxon No. 2817](http://scriptorium.cos.uni-heidelberg.de/php/taxon_details.php?tx=2817) |  |
| AY614425 | *Guzmania herrerae* H. Luther & W.J. Kress. | -24.1 ^a^ |  |  |  | Epiphyte | [WRHP Taxon No. 2825](http://scriptorium.cos.uni-heidelberg.de/php/taxon_details.php?tx=2825) |  |
| AY614417 | *Guzmania melinonis* Regel. | -26 ^a^ |  |  |  | Epiphyte | [WRHP Taxon No. 2826](http://scriptorium.cos.uni-heidelberg.de/php/taxon_details.php?tx=2826) |  |
| AY614420 | *Guzmania monostachia* (L.) Rusby ex Mez var. *monostachia* | -26.7 ^f^ |  |  |  | Epiphyte, terrestial, lithophyte | [WRHP Taxon No. 2044](http://scriptorium.cos.uni-heidelberg.de/php/taxon_details.php?tx=2044) |  |
|  |  | -26.5 ^d^ |  |  |  |  |  |  |
|  |  | -25.1 ^a^ (Average -26.1) |  |  |  |  |  |  |
| AY614427 | *Guzmania multiflora* (André) André ex Mez. | -24 ^f^ |  |  |  | Epiphyte | [WRHP Taxon No. 1596](http://scriptorium.cos.uni-heidelberg.de/php/taxon_details.php?tx=1596) |  |
|  |  | -22.4 ^a^ (Average -22.4) |  |  |  |  |  |  |
| AY614424 | *Guzmania musaica* (Linden & André) Mez var. *musaica* | -24.7 ^a^ |  |  |  | Epiphyte | [WRHP Taxon No. 2494](http://scriptorium.cos.uni-heidelberg.de/php/taxon_details.php?tx=2494) |  |
| AY614431 | *Guzmania patula* Mez & Wercklé. | -28.7 ^a^ |  |  |  | Epiphyte, terrestial, lithophyte | [WRHP Taxon No. 2828](http://scriptorium.cos.uni-heidelberg.de/php/taxon_details.php?tx=2828) |  |
| AY614430 | *Guzmania rhonhofiana* Harms. | -28.1 ^a^ |  |  |  | Epiphyte | [WRHP Taxon No. 2829](http://scriptorium.cos.uni-heidelberg.de/php/taxon_details.php?tx=2829) |  |
| AY614421 | *Guzmania variegata* L.B. Sm. | -26.4 ^a^ |  |  |  | Epiphyte, terrestial | [WRHP Taxon No. 2827](http://scriptorium.cos.uni-heidelberg.de/php/taxon_details.php?tx=2827) |  |
| AY614422 | *Guzmania wittmackii* (André) André ex Mez. | -29.1 ^a^ |  |  |  | Epiphyte | [WRHP Taxon No. 2830](http://scriptorium.cos.uni-heidelberg.de/php/taxon_details.php?tx=2830) |  |
| L19974 | *Hechtia montana* Brand. | -13.1 ^a^ |  |  |  | Lithophyte | [WRHP Taxon No. 2831](http://scriptorium.cos.uni-heidelberg.de/php/taxon_details.php?tx=2831) |  |
| KC123372 | *Lapanthus* duartei (L.B. Sm.) Louzada & Versieux. | -28.9 ^a^ |  |  |  | Terrestrial, lithophyte | [WRHP Taxon No. 2832](http://scriptorium.cos.uni-heidelberg.de/php/taxon_details.php?tx=2832) |  |
| AY614385 | *Lindmania* guianensis (Beer) Mez. | -24.7 ^a^ |  |  |  | Terrestrial | [WRHP Taxon No. 2833](http://scriptorium.cos.uni-heidelberg.de/php/taxon_details.php?tx=2833) |  |
| HQ182439 | *Navia saxicola* L.B. Sm. | -26.8 ^a^ |  |  |  | Lithophyte | [WRHP Taxon No. 2834](http://scriptorium.cos.uni-heidelberg.de/php/taxon_details.php?tx=2834) |  |
| KC123377 | *Neoglaziovia variegata* (Arruda) Mez. | -9.4 ^a^ |  |  |  | Terrestrial, lithophyte | [WRHP Taxon No. 2835](http://scriptorium.cos.uni-heidelberg.de/php/taxon_details.php?tx=2835) |  |
| KC123378 | *Ochagavia elegans* Philippi | -22.4 ^a^ |  |  |  | Lithophyte |  |  |
| KC123379 | *Orthophytum disjunctum* L.B. Sm. | -15.1 ^a^ |  |  |  | Lithophyte | [WRHP Taxon No. 2836](http://scriptorium.cos.uni-heidelberg.de/php/taxon_details.php?tx=2836) |  |
| HQ182445 | *Pitcairnia feliciana* (A. Chev.) Harms & Mildbraed. | -23.8 ^a^ |  |  |  | Lithophyte | [WRHP Taxon No. 2837](http://scriptorium.cos.uni-heidelberg.de/php/taxon_details.php?tx=2837) |  |
| AY614387 | *Pitcairnia punicea* Scheidw. | -30.1 ^a^ |  |  |  | Terrestrial, lithophyte | [WRHP Taxon No. 2838](http://scriptorium.cos.uni-heidelberg.de/php/taxon_details.php?tx=2838) |  |
|  |  | -29.7 ^a^ (Average -29.9) |  |  |  |  |  |  |
| KC123382 | *Puya chilensis* Molina | -22.1 ^a^ |  |  |  | Terrestrial | [WRHP Taxon No. 2839](http://scriptorium.cos.uni-heidelberg.de/php/taxon_details.php?tx=2839) |  |
|  |  | -18.6 ^a^ |  |  |  |  |  |  |
|  |  | -16.3 ^a^ (Average -19) |  |  |  |  |  |  |
| L19973 | *Puya dyckioides* Baker | -22.5 ^a^ |  |  |  | Terrestrial | [WRHP Taxon No. 2840](http://scriptorium.cos.uni-heidelberg.de/php/taxon_details.php?tx=2840) |  |
|  |  | -21.8 ^a^ (Average -22.2) |  |  |  |  |  |  |
| AY614388 | *Puya laxa* L.B.Smith | -15.8 ^f^ | 6.2 ± 0.5 | 3.8 ± 0.2 | 404.5 ± 27.7 | Terrestrial | [WRHP Taxon No. 2199](http://scriptorium.cos.uni-heidelberg.de/php/taxon_details.php?tx=2199) |  |
| AF206814 | *Puya raimondii* Harms. | -22.2 ^a^ |  |  |  | Terrestrial | [WRHP Taxon No. 2824](http://scriptorium.cos.uni-heidelberg.de/php/taxon_details.php?tx=2824) |  |
| AY614450 | *Racinaea elegans* (L.B. Sm.) M.A. Spencer & L.B. Sm. | -26.3 ^a^ |  |  |  | Epiphyte | [WRHP Taxon No. 2841](http://scriptorium.cos.uni-heidelberg.de/php/taxon_details.php?tx=2841) |  |
| AY614449 | *Racinaea ropalocarpa* (André) M.A. Spencer & L.B. Sm. | -24 ^a^ |  |  |  | Epiphyte | [WRHP Taxon No. 2842](http://scriptorium.cos.uni-heidelberg.de/php/taxon_details.php?tx=2842) |  |
| AY614451 | *Racinaea seemannii* (Baker) M.A. Spencer & L.B. Sm. | -30.2 ^a^ |  |  |  | Epiphyte | [WRHP Taxon No. 2843](http://scriptorium.cos.uni-heidelberg.de/php/taxon_details.php?tx=2843) |  |
| AY614448 | *Racinaea spiculosa* (Griseb.) M.A. Spencer & L.B. Sm. var. *spiculosa* | -25.9 ^a^ |  |  |  | Epiphyte | [WRHP Taxon No. 2279](http://scriptorium.cos.uni-heidelberg.de/php/taxon_details.php?tx=2279) |  |
| FM211061 | *Tillandsia achyrostachys* E. Morren ex Baker | -14.7 ^a^ |  |  |  | Epiphyte | [WRHP Taxon No. 2844](http://scriptorium.cos.uni-heidelberg.de/php/taxon_details.php?tx=2844) |  |
| AY614497 | *Tillandsia aeranthos* (Loisel.) L.B. Sm. | -16.1 ^a^ |  |  |  | Epiphyte | [WRHP Taxon No. 2845](http://scriptorium.cos.uni-heidelberg.de/php/taxon_details.php?tx=2845) |  |
| AY614454 | *Tillandsia andrieuxii* (Mez) L.B.Sm. | -23.4 ^a^ |  |  |  | Epiphyte | [WRHP Taxon No. 2204](http://scriptorium.cos.uni-heidelberg.de/php/taxon_details.php?tx=2204) |  |
| AY614490 | *Tillandsia argentina* C.H.Wright | -13.2 ^a^ | 3.7 ± 0.4 | 1.2 ± 0.1 | 291.9 ± 44.9 | Epiphyte, lithophyte | [WRHP Taxon No. 2205](http://scriptorium.cos.uni-heidelberg.de/php/taxon_details.php?tx=2205) |  |
| AY614480 | *Tillandsia baliophylla* Harms | -23.1 ^a^ |  |  |  | Epiphyte, terrestrial, lithophyte | [WRHP Taxon No. 2847](http://scriptorium.cos.uni-heidelberg.de/php/taxon_details.php?tx=2847) |  |
| AY614445 | *Tillandsia barclayana* Baker | -18.5 ^a^ |  |  |  | Epiphyte | [WRHP Taxon No. 1554](http://scriptorium.cos.uni-heidelberg.de/php/taxon_details.php?tx=1554) |  |
| AY614442 | *Tillandsia barthlottii* Rauh | -22.6 ^a^ |  |  |  | Epiphyte | [WRHP Taxon No. 818](http://scriptorium.cos.uni-heidelberg.de/php/taxon_details.php?tx=818) |  |
| KJ773949 | *Tillandsia bartramii* Elliott | -14 ^a^ |  |  |  | Epiphyte | [WRHP Taxon No. 2848](http://scriptorium.cos.uni-heidelberg.de/php/taxon_details.php?tx=2848) |  |
| AY614499 | *Tillandsia bergeri* Mez. | -13.5 ^a^ | 4.2 ± 0.2 | 0.5 ± 0.1 | 122.3 ± 7.1 | Epiphyte | [WRHP Taxon No. 2206](http://scriptorium.cos.uni-heidelberg.de/php/taxon_details.php?tx=2206) |  |
| AY614489 | *Tillandsia bermejoensis* H.Hrom. ex Rauh | -13.8 ^a^ | 4.3 ± 0.2 | 1.0 ± 0.1 | 164.8 ± 6.6 | Epiphyte, lithophyte | [WRHP Taxon No. 2207](http://scriptorium.cos.uni-heidelberg.de/php/taxon_details.php?tx=2207) |  |
| AY614473 | *Tillandsia biflora* Ruiz & Pav. | -25.1 ^a^ | 5.7 ± 0.2 | 0.2 ± 0.1 | 42.2 ± 1.2 | Epiphyte | [WRHP Taxon No. 946](http://scriptorium.cos.uni-heidelberg.de/php/taxon_details.php?tx=946) |  |
| AY614471 | *Tillandsia brachyphylla* Baker | -14.3 ^a^ | 5.1 ± 0.2 | 0.7 ± 0.1 | 97.2 ± 8.9 | Epiphyte, lithophyte | [WRHP Taxon No. 2208](http://scriptorium.cos.uni-heidelberg.de/php/taxon_details.php?tx=2208) |  |
| AY614479 | *Tillandsia brevilingua* Mez ex Harms | -28.2 ^a^ |  |  |  | Epiphyte | [WRHP Taxon No. 2849](http://scriptorium.cos.uni-heidelberg.de/php/taxon_details.php?tx=2849) |  |
| AY614436 | *Tillandsia cacticola* L.B. Sm. | -17.2 ^a^ |  |  |  | Epiphyte | [WRHP Taxon No. 2103](http://scriptorium.cos.uni-heidelberg.de/php/taxon_details.php?tx=2103) |  |
| AY614464 | *Tillandsia caput-medusae* E. Morren | -15.4 ^a^ | 7.8 ± 0.3 | 1.7 ± 0.2 | 176.9 ± 39.3 | Epiphyte | [WRHP Taxon No. 1319](http://scriptorium.cos.uni-heidelberg.de/php/taxon_details.php?tx=1319) |  |
| AY614455 | *Tillandsia carlos-hankii* Matuda | -20.8 ^a^ |  |  |  | Epiphyte | [WRHP Taxon No. 2850](http://scriptorium.cos.uni-heidelberg.de/php/taxon_details.php?tx=2850) |  |
| AY614492 | *Tillandsia caulescens* Brongn. ex Baker | -14.8 ^a^ |  |  |  | Epiphyte, lithophyte | [WRHP Taxon No. 720](http://scriptorium.cos.uni-heidelberg.de/php/taxon_details.php?tx=720) |  |
| AY614468 | *Tillandsia coinaensis* Ehlers | -23.1 ^a^ | 5.6 ± 0.1 | 0.6 ± 0.1 | 81.0 ± 5.2 | Epiphyte, terrestrial, lithophyte | [WRHP Taxon No. 1607](http://scriptorium.cos.uni-heidelberg.de/php/taxon_details.php?tx=1607) |  |
| AY614481 | *Tillandsia demissa* L.B. Sm. | -19.3 ^a^ |  |  |  | Lithophyte | [WRHP Taxon No. 2851](http://scriptorium.cos.uni-heidelberg.de/php/taxon_details.php?tx=2851) |  |
| AY614493 | *Tillandsia didisticha* (E. Morren) Baker | -16.7 ^a^ | 4.0 ± 0.4 | 1.1 ± 0.1 | 213.5 ± 12.5 | Epiphyte, lithophyte | [WRHP Taxon No. 2209](http://scriptorium.cos.uni-heidelberg.de/php/taxon_details.php?tx=2209) |  |
| AY614434 | *Tillandsia disticha* Kunth | -17.8 ^a^ |  |  |  | Epiphyte, terrestrial | [WRHP Taxon No. 1597](http://scriptorium.cos.uni-heidelberg.de/php/taxon_details.php?tx=1597) |  |
| AY614438 | *Tillandsia dodsonii* L.B. Sm. | -23.1 ^a^ |  |  |  | Epiphyte | [WRHP Taxon No. 2047](http://scriptorium.cos.uni-heidelberg.de/php/taxon_details.php?tx=2047) |  |
| AY614485 | *Tillandsia duratii* Vis. var*. duratii* | -12.8 ^a^ |  |  |  | Epiphyte | [WRHP Taxon No. 2852](http://scriptorium.cos.uni-heidelberg.de/php/taxon_details.php?tx=2852) |  |
| L19971 | *Tillandsia elizabethae* Rauh | -13.5 ^a^ |  |  |  | Epiphyte | [WRHP Taxon No. 2441](http://scriptorium.cos.uni-heidelberg.de/php/taxon_details.php?tx=2441) |  |
| FM211065 | *Tillandsia exserta* Fernald | -13.6 ^a^ |  |  |  | Epiphyte | [WRHP Taxon No. 2853](http://scriptorium.cos.uni-heidelberg.de/php/taxon_details.php?tx=2853) |  |
| FM211066 | *Tillandsia fasciculata* Swartz var. *fasciculata* | -14.2 ^e^ | 5.2 ± 0.2 | 2.1 ± 0.1 | 292.2 ± 30.0 | Epiphyte | [WRHP Taxon No. 1143](http://scriptorium.cos.uni-heidelberg.de/php/taxon_details.php?tx=1143) |  |
|  |  | -19.9 ^a^ (Average -17.5) |  |  |  |  |  |  |
| AY614482 | *Tillandsia fendleri* Griseb. var*. fendleri* | -31.8 ^d^ | 5.9 ± 0.3 | 0.4 ± 0.1 | 60.1 ± 0.9 | Epiphyte | [WRHP Taxon No. 1148](http://scriptorium.cos.uni-heidelberg.de/php/taxon_details.php?tx=1148) |  |
|  |  | -23 ^a^ (Average -27.4) |  |  |  |  |  |  |
| AY614484 | *Tillandsia funebris* A. Cast. | -16.2 ^a^ |  |  |  | Epiphyte, lithophyte | [WRHP Taxon No. 2854](http://scriptorium.cos.uni-heidelberg.de/php/taxon_details.php?tx=2854) |  |
| AY614470 | *Tillandsia gardneri* Lindl. var. *gardneri* | -14.7 ^d^ |  |  |  | Epiphyte, terrestrial, lithophyte | [WRHP Taxon No. 1013](http://scriptorium.cos.uni-heidelberg.de/php/taxon_details.php?tx=1013) |  |
|  |  | -12 ^a^ (Average -13.35) |  |  |  |  |  |  |
| AY614459 | *Tillandsia guatemalensis* L.B. Sm. | -25.3 ^a^ | 4.2 ± 0.3 | 0.3 ± 0.1 | 61.9 ± 8.1 | Epiphyte | [WRHP Taxon No. 1750](http://scriptorium.cos.uni-heidelberg.de/php/taxon_details.php?tx=1750) |  |
| AY614477 | *Tillandsia heterophylla* E. Morren | -28.1 ^a^ |  |  |  | Epiphyte | [WRHP Taxon No. 2855](http://scriptorium.cos.uni-heidelberg.de/php/taxon_details.php?tx=2855) |  |
| AY614472 | *Tillandsia heubergeri* Ehlers | -14.8 ^a^ |  |  |  | Epiphyte, lithophyte | [WRHP Taxon No. 2856](http://scriptorium.cos.uni-heidelberg.de/php/taxon_details.php?tx=2856) |  |
| AY614495 | *Tillandsia ixioides* Griseb. | -14.2 ^a^ | 3.4 ± 0.2 | 1.2 ± 0.1 | 299.2 ± 42.9 | Epiphyte | [WRHP Taxon No. 2210](http://scriptorium.cos.uni-heidelberg.de/php/taxon_details.php?tx=2210) |  |
| AY614463 | *Tillandsia juncea* (Ruiz & Pav.) Poir. | -14.5 ^a^ |  |  |  | Epiphyte, lithophyte | [WRHP Taxon No. 1506](http://scriptorium.cos.uni-heidelberg.de/php/taxon_details.php?tx=1506) |  |
|  |  | -13.4 ^d^ |  |  |  |  |  |  |
|  |  | -13.3 ^a^ (Average -13.7) |  |  |  |  |  |  |
| AY614474 | *Tillandsia latifolia* var. *divaricata* (Benth.) Mez | -14.3 ^a^ | 4.8 ± 0.2 | 0.8 ± 0.1 | 126.3 ± 24.4 | Epiphyte, terrestrial, lithophyte | [WRHP Taxon No. 2102](http://scriptorium.cos.uni-heidelberg.de/php/taxon_details.php?tx=2102) |  |
| FM211067 | *Tillandsia lepidosepala* L.B. Sm. | -13.3 ^a^ |  |  |  | Epiphyte | [WRHP Taxon No. 1758](http://scriptorium.cos.uni-heidelberg.de/php/taxon_details.php?tx=1758) |  |
| AY614435 | *Tillandsia marconae* W. Till & Vitek | -15.3 ^a^ |  |  |  | Terrestrial | [WRHP Taxon No. 2857](http://scriptorium.cos.uni-heidelberg.de/php/taxon_details.php?tx=2857) |  |
| FM211068 | *Tillandsia matudae* L.B. Sm. | -16.8 ^a^ |  |  |  | Epiphyte | [WRHP Taxon No. 2858](http://scriptorium.cos.uni-heidelberg.de/php/taxon_details.php?tx=2858) |  |
| AY614478 | *Tillandsia multicaulis* Steud. | -27.1 ^a^ | 3.3 ± 1.0 | 0.3 ± 0.1 | 196.1 ± 138.3 | Epiphyte | [WRHP Taxon No. 1152](http://scriptorium.cos.uni-heidelberg.de/php/taxon_details.php?tx=1152) |  |
| AY614437 | *Tillandsia narthecioides* C. Presl | -23.5 ^a^ |  |  |  | Epiphyte | [WRHP Taxon No. 2130](http://scriptorium.cos.uni-heidelberg.de/php/taxon_details.php?tx=2130) |  |
| AY614452 | *Tillandsia paniculata* (L.) L. | -15.4 ^a^ |  |  |  | Epiphyte, lithophyte | [WRHP Taxon No. 2859](http://scriptorium.cos.uni-heidelberg.de/php/taxon_details.php?tx=2859) |  |
| FN550874 | *Tillandsia paucifolia* Baker | -12.6 ^a^ |  |  |  | Epiphyte | [WRHP Taxon No. 2860](http://scriptorium.cos.uni-heidelberg.de/php/taxon_details.php?tx=2860) |  |
| AY614494 | *Tillandsia pohliana* Mez | -13.3 ^a^ |  |  |  | Epiphyte | [WRHP Taxon No. 976](http://scriptorium.cos.uni-heidelberg.de/php/taxon_details.php?tx=976) |  |
| AY614453 | *Tillandsia punctulata* Schltdl. & Cham. | -24.5 ^a^ | 5.2 ± 0.2 | 0.9 ± 0.1 | 132.3 ± 13.1 | Epiphyte | [WRHP Taxon No. 1567](http://scriptorium.cos.uni-heidelberg.de/php/taxon_details.php?tx=1567) |  |
| AY614467 | *Tillandsia rauhii* L.B. Sm.var. *rauhii* | -20.8 ^a^ |  |  |  | Lithophyte | [WRHP Taxon No. 794](http://scriptorium.cos.uni-heidelberg.de/php/taxon_details.php?tx=794) |  |
| AY614461 | *Tillandsia remota* Wittm. | -14.5 ^a^ |  |  |  | Epiphyte | [WRHP Taxon No. 2861](http://scriptorium.cos.uni-heidelberg.de/php/taxon_details.php?tx=2861) |  |
| AY614405 | *Tillandsia singularis* Mez & Wercklé | -26.9 ^a^ |  |  |  | Epiphyte | [WRHP Taxon No. 2862](http://scriptorium.cos.uni-heidelberg.de/php/taxon_details.php?tx=2862) |  |
| AY614496 | *Tillandsia stricta* Sol. ex Sims var. *stricta* | -14.9 ^d^ |  |  |  | Epiphyte | [WRHP Taxon No. 1014](http://scriptorium.cos.uni-heidelberg.de/php/taxon_details.php?tx=1014) |  |
|  |  | -14.9 ^a^ (Average -14.9) |  |  |  |  |  |  |
| AY614439 | *Tillandsia tectorum* E. Morren | -14.2 ^a^ |  |  |  | Epiphyte, terrestrial, lithophyte | [WRHP Taxon No. 1598](http://scriptorium.cos.uni-heidelberg.de/php/taxon_details.php?tx=1598) |  |
| AY614498 | *Tillandsia tenuifolia* L.var. *tenuifolia* | -14.6 ^a^ |  |  |  | Epiphyte, lithophyte | [WRHP Taxon No. 1572](http://scriptorium.cos.uni-heidelberg.de/php/taxon_details.php?tx=1572) |  |
| AY614487 | *Tillandsia usneoides* (L.) L. | -19.8 ^d^ |  |  |  | Epiphyte | [WRHP Taxon No. 1504](http://scriptorium.cos.uni-heidelberg.de/php/taxon_details.php?tx=1504) |  |
|  |  | -18.6 ^a^ |  |  |  |  |  |  |
|  |  | -18.6 ^g^ (Average -19) |  |  |  |  |  |  |
| AY614456 | *Tillandsia utriculata* L. fo. *utriculata* | -11.4 ^a^ |  |  |  | Epiphyte, lithophyte | [WRHP Taxon No. 2863](http://scriptorium.cos.uni-heidelberg.de/php/taxon_details.php?tx=2863) |  |
|  |  | -11.2 ^d^ (Average -11.3) |  |  |  |  |  |  |
| AY614447 | *Tillandsia venusta* Mez & Wercklé | -27.2 ^a^ |  |  |  | Epiphyte | [WRHP Taxon No. 2864](http://scriptorium.cos.uni-heidelberg.de/php/taxon_details.php?tx=2864) |  |
| AY614432 | *Tillandsia viridiflora* (Beer) Baker var. *viridiflora* | -24.8 ^a^ |  |  |  | Epiphyte | [WRHP Taxon No. 2865](http://scriptorium.cos.uni-heidelberg.de/php/taxon_details.php?tx=2865) |  |
| AY614433 | *Tillandsia wagneriana* L.B. Sm. | -30.9 ^a^ |  |  |  | Epiphyte | [WRHP Taxon No. 2866](http://scriptorium.cos.uni-heidelberg.de/php/taxon_details.php?tx=2866) |  |
| AY614444 | *Tillandsia werneriana* J.R. Grant | -19.7 ^a^ |  |  |  | Epiphyte, lithophyte | [WRHP Taxon No. 797](http://scriptorium.cos.uni-heidelberg.de/php/taxon_details.php?tx=797) |  |
| AY614491 | *Tillandsia xiphioides* Ker Gawl. var. *xiphioides* | -14.3 ^a^ |  |  |  | Epiphyte, lithophyte | [WRHP Taxon No. 2868](http://scriptorium.cos.uni-heidelberg.de/php/taxon_details.php?tx=2868) |  |
|  |  | -12.4 ^a^ (Average -13.35) |  |  |  |  |  |  |
| AY614399 | *Vriesea carinata* Wawra | -26.9 ^a^ |  |  |  | Epiphyte | [WRHP Taxon No. 2869](http://scriptorium.cos.uni-heidelberg.de/php/taxon_details.php?tx=2869) |  |
| AY614407 | *Vriesea chrysostachys* E. Morren | -25.1 ^d^ |  |  |  | Epiphyte, terrestrial, lithophyte | [WRHP Taxon No. 2870](http://scriptorium.cos.uni-heidelberg.de/php/taxon_details.php?tx=2870) |  |
|  |  | -22.3 ^a^ (Average -23.7) |  |  |  |  |  |  |
| AY614401 | *Vriesea correia-araujoi* E. Pereira & L.A. Penna | -24.6 ^a^ |  |  |  | Epiphyte | [WRHP Taxon No. 2871](http://scriptorium.cos.uni-heidelberg.de/php/taxon_details.php?tx=2871) |  |
| AY614403 | *Vriesea jonghei* (Libon ex K. Koch) E. Morren | -25 ^a^ |  |  |  | Epiphyte, terrestrial, lithophyte | [WRHP Taxon No. 2872](http://scriptorium.cos.uni-heidelberg.de/php/taxon_details.php?tx=2872) |  |
| AY614410 | *Vriesea monstrum* (Mez) L.B. Sm. | -26.3 ^a^ |  |  |  | Epiphyte, terrestrial | [WRHP Taxon No. 2873](http://scriptorium.cos.uni-heidelberg.de/php/taxon_details.php?tx=2873) |  |
|  |  | -25.9 ^f^ (Average -26.1) |  |  |  |  |  |  |
| AY614406 | *Vriesea ospinae* H. Luther var. *ospinae* | -28 ^f^ |  |  |  | - | [WRHP Taxon No. 2874](http://scriptorium.cos.uni-heidelberg.de/php/taxon_details.php?tx=2874) |  |
|  |  | -23.6 ^a^ (Average -25.8) |  |  |  |  |  |  |
| AM110253 | *Vriesea psittacina* (Hook.) Lindl. | -31.2 ^a^ |  |  |  | Epiphyte, lithophyte | [WRHP Taxon No. 2875](http://scriptorium.cos.uni-heidelberg.de/php/taxon_details.php?tx=2875) |  |
| AY614402 | *Vriesea saundersii* (Carrière) E. Morren | -25.2 ^a^ |  |  |  | Epiphyte, terrestrial, lithophyte | [WRHP Taxon No. 2876](http://scriptorium.cos.uni-heidelberg.de/php/taxon_details.php?tx=2876) |  |
| AY614411 | *Vriesea splendens* (Brongn.) Lem. var. *splendens* | -34.8 ^d^ |  |  |  | Epiphyte, terrestrial | [WRHP Taxon No. 2877](http://scriptorium.cos.uni-heidelberg.de/php/taxon_details.php?tx=2877) |  |
|  |  | -30.1 ^a^ (Average -32.45) |  |  |  |  |  |  |
| AY614409 | *Vriesea zamorensis* L.B. Sm. | -27.7 ^a^ |  |  |  | Epiphyte | [WRHP Taxon No. 2128](http://scriptorium.cos.uni-heidelberg.de/php/taxon_details.php?tx=2128) |  |
| AY614415 | *Werauhia insignis* (Mez) W. Till, Barfuss & R. Samuel | -28.9 ^a^ |  |  |  | Epiphyte, terrestrial | [WRHP Taxon No. 2878](http://scriptorium.cos.uni-heidelberg.de/php/taxon_details.php?tx=2878) |  |
| AY614414 | *Werauhia ororiensis* (Mez) J.R. Grant | -25.6 ^a^ |  |  |  | Epiphyte, terrestrial, lithophyte | [WRHP Taxon No. 2879](http://scriptorium.cos.uni-heidelberg.de/php/taxon_details.php?tx=2879) |  |
| AY614413 | *Werauhia ringens* (Griseb.) J.R. Grant | -27.6 ^a^ |  |  |  | Epiphyte | [WRHP Taxon No. 2880](http://scriptorium.cos.uni-heidelberg.de/php/taxon_details.php?tx=2880) |  |

[99] Griffiths H, Smith JAC. Photosynthetic pathways in the Bromeliaceae of Trinidad: relations between life-forms, habitat preference and the occurrence of CAM. Oecologia. 1983;60:176–184.

[100] Zotz G, Ziegler H. The occurrence of crassulacean acid methabolism among vascular epiphytes from central panama. New Phytol. 1997;137:223–219

[101] Pierce S, Winter K., Griffiths H. The role of CAM in high rainfall cloud forests: an in situ comparison of photosynthetic pathways in Bromeliaceae. 2002;25:1181–1189.

[102] Smith B.N.; Epstein S. Two categories of 13C/12C ratios for higher plants. Plant physiology. 1971;47:380–384.

[103] Holtum JA, Hancock LP, Edwards EJ, Crisp MD, Crayn DM, Sage R, Winter K. Australia lacks stem succulents but is it depauperate in plants with crassulacean acid metabolism (CAM)?. Curr Opin Plant Biol. 2016;31:109–117.

[104] Hynson NA, Preiss K, Gebauer G. Is it better to give than to receive? A stable isotope perspective on orchid–fungal carbon transport in the green orchid species *Goodyera repens* and *Goodyera oblongifolia*. New Phytol. 2009;182:8–11.
