# Supplementary material for: Exploring molecular evolution of Rubisco in C3 and CAM Orchidaceae and Bromeliaceae
Source: BMC Evol Biol. 2020 Jan 22;20:11. doi: 10.1186/s12862-019-1551-8 (PMC6977233; doi:10.1186/s12862-019-1551-8)
Supplement: Supplementary file 2 — Additional file 2: Table S2. Mean ± S.E. (n = 4) for the leaf mass per area (LMA), the leaf thickness and the leaf fresh to dry weight ratio (FW/DW) of C3, weak CAM and strong CAM for orchids and bromeliads. Values for the individual species are shown in Table 1 and Additional file 1: Table S1. Different letters denote statistically significant differences among metabolic types through Duncan test (p < 0.05). [file 12862_2019_1551_MOESM2_ESM.docx]

**Additional file 2: Table S2.** Mean ± S.E. (*n* = 4) for the leaf mass per area (LMA), the leaf thickness and the leaf fresh to dry weight ratio (FW/DW) of C_3_, weak CAM and strong CAM for orchids and bromeliads. Values for the individual species are shown in Table 1 and Additional file 1: Table S1. Different letters denote statistically significant differences among metabolic types through Duncan test (*p* < 0.05).

|  | Orchids | | |
| --- | --- | --- | --- |
|  | LMA (g m^–2^) | Leaf thickness (mm) | Leaf FW/DW |
| C_3_ | 78.4 ± 3.8^a^ | 0.7 ± 0.1^a^ | 6.6 ± 0.4^a^ |
| Weak CAM | 134.5 ± 8.5^b^ | 1.4 ± 0.2^b^ | 6,3 ± 1.2^a^ |
| Strong CAM | 185.4 ± 10.5^c^ | 1.6 ± 0.1^b^ | 6.9 ± 0.4^a^ |
|  | Bromeliads | | |
|  | LMA (g m^–2^) | Leaf thickness (mm) | Leaf FW/DW |
| C_3_ | 117.1 ± 15.2^a^ | 0.7 ± 0.1^a^ | 4.9 ± 0.2^a^ |
| Strong CAM | 190.2 ± 12.3^b^ | 1.3 ± 0.1^b^ | 4.6 ± 0.1^a^ |
