# Supplementary material for: Exploring molecular evolution of Rubisco in C3 and CAM Orchidaceae and Bromeliaceae
Source: BMC Evol Biol. 2020 Jan 22;20:11. doi: 10.1186/s12862-019-1551-8 (PMC6977233; doi:10.1186/s12862-019-1551-8)
Supplement: Supplementary file 3 — Additional file 3: Table S3. Coevolving groups of residues detected within the L-subunit of Rubisco within orchids and bromeliads. [file 12862_2019_1551_MOESM3_ESM.docx]

**Additional file 3: Table S3.** Coevolving groups of residues detected within the L-subunit of Rubisco among orchids and bromeliads.

|  | Orchids | | | | | | | | | | | | | | |
| --- | --- | --- | --- | --- | --- | --- | --- | --- | --- | --- | --- | --- | --- | --- | --- |
| Group 1 | 26 | 28 | 328 | 334 | 443 | 447 | 449 | 461 | 466 | 468 | 470 | 475 | 477 | 478 | 479 |
| Group 2 | 26 | 28 | 439 | 443 | 449 | 461 | 466 | 468 | 470 | 475 | 477 | 478 | 479 |  |  |
| Group 3 | 26 | 28 | 265 | 439 | 443 | 447 | 449 | 461 | 466 | 468 | 470 | 475 | 477 | 478 | 479 |
| Group 4 | 26 | 28 | 279 | 443 | 447 | 449 | 461 | 466 | 468 | 470 | 475 | 477 | 478 | 479 |  |
| Group 5 | 33 | 340 | 353 | 359 |  |  |  |  |  |  |  |  |  |  |  |
| Group 6 | 328 | 340 | 359 |  |  |  |  |  |  |  |  |  |  |  |  |
| Group 7 | 341 | 439 | 475 |  |  |  |  |  |  |  |  |  |  |  |  |
| Group 8 | 439 | 466 | 475 |  |  |  |  |  |  |  |  |  |  |  |  |
| Group 9 | 341 | 466 | 475 |  |  |  |  |  |  |  |  |  |  |  |  |
| Group 10 | 340 | 353 | 359 |  |  |  |  |  |  |  |  |  |  |  |  |
| Group 11 | 265 | 279 |  |  |  |  |  |  |  |  |  |  |  |  |  |

| Bromeliads | | | | | | | | | | | | | | | | | | | | |
| --- | --- | --- | --- | --- | --- | --- | --- | --- | --- | --- | --- | --- | --- | --- | --- | --- | --- | --- | --- | --- |
| Group 1 | 28 | 91 | 97 | 116 | 142 | 143 | 219 | 225 | 245 | 251 | 255 | 262 | 270 | 279 | 407 | 449 | 464 | 468 | 470 | 478 |
| Group 2 | 449 | 478 |  |  |  |  |  |  |  |  |  |  |  |  |  |  |  |  |  |  |
