# Supplementary material for: Exploring molecular evolution of Rubisco in C3 and CAM Orchidaceae and Bromeliaceae
Source: BMC Evol Biol. 2020 Jan 22;20:11. doi: 10.1186/s12862-019-1551-8 (PMC6977233; doi:10.1186/s12862-019-1551-8)
Supplement: Supplementary file 4 — Additional file 4: Table S4. Integrative view of the Rubisco L-subunit variable sites under positive selection, coevolving and resolved with DT model as a function of external variables (δ13C and habitat preference). [file 12862_2019_1551_MOESM4_ESM.docx]

**Additional file 4: Table S4.** Integrative view of the Rubisco L-subunit variable sites under positive selection, coevolving and resolved with DT model as a function of external variables (δ^13^C and habitat preference). Functional interfaces in Rubisco structure are indicated according to [105–109]: *RA*: Rubisco Activase, *DD*: dimer-dimer, *S*: Small Subunit, *ID*: Intra-dimer, *AS*: Active Site. Observed amino acid replacements are shown for each site.

|  | Orchids | | | | | | | | | | | | | | | | | | | | | | | | | | | | | | |
| --- | --- | --- | --- | --- | --- | --- | --- | --- | --- | --- | --- | --- | --- | --- | --- | --- | --- | --- | --- | --- | --- | --- | --- | --- | --- | --- | --- | --- | --- | --- | --- |
| Sites | 26 | 28 | 33 | 89 | 142 | 224 | 225 | 251 | 265 | 279 | 282 | 284 | 328 | 334 | 340 | 341 | 353 | 359 | 375 | 439 | 443 | 447 | 449 | 461 | 466 | 468 | 470 | 475 | 477 | 478 | 479 |
| Positive selection |  |  |  |  |  |  |  |  |  |  |  |  |  |  |  |  |  |  |  |  |  |  |  |  |  |  |  |  |  |  |  |
| Decision Tree |  |  |  |  |  |  |  |  |  |  |  |  |  |  |  |  |  |  |  |  |  |  |  |  |  |  |  |  |  |  |  |
| Coevolution |  |  |  |  |  |  |  |  |  |  |  |  |  |  |  |  |  |  |  |  |  |  |  |  |  |  |  |  |  |  |  |
| Interface |  |  |  | *RA* | *DD* | *S* | *S* |  |  |  |  |  | *AS* | *ID/ AS* |  |  |  |  |  |  |  |  |  | *ID* | *ID* | *ID* | *ID* |  |  |  |  |
| Amino acids | T | D | D | V | T | A | L | I | V | S | H | C | A | K | E | M | F | S | L | R | D | E | S | I | K | E | E | V | T | L | D |
|  | A | A | S | A | P | S | I | M | I | T | Q | S | S | E | D | V | Y | N | I | L | E | K | C | V | T | D | D | L | K | D | P |
|  |  |  |  | G |  |  |  | V |  |  |  |  | P |  |  | L |  |  |  |  |  |  | T |  |  | N |  | P | Q | E | T |
|  |  |  |  | S |  |  |  |  |  |  |  |  |  |  |  |  |  |  |  |  |  |  | A |  |  |  |  |  |  |  | K |
|  |  |  |  | P |  |  |  |  |  |  |  |  |  |  |  |  |  |  |  |  |  |  |  |  |  |  |  |  |  |  |  |
|  | Bromeliads | | | | | | | | | | | | | | | | | | | |  |  |  |  |  |  |  |  |  |  |  |
| Sites | 28 | 91 | 97 | 116 | 142 | 143 | 219 | 225 | 245 | 251 | 255 | 262 | 270 | 279 | 407 | 449 | 464 | 468 | 470 | 478 |  |  |  |  |  |  |  |  |  |  |  |
| Positive selection |  |  |  |  |  |  |  |  |  |  |  |  |  |  |  |  |  |  |  |  |  |  |  |  |  |  |  |  |  |  |  |
| Decision Tree |  |  |  |  |  |  |  |  |  |  |  |  |  |  |  |  |  |  |  |  |  |  |  |  |  |  |  |  |  |  |  |
| Coevolution |  |  |  |  |  |  |  |  |  |  |  |  |  |  |  |  |  |  |  |  |  |  |  |  |  |  |  |  |  |  |  |
| Interface |  | *RA* |  | *ID* | *DD* | *DD* | *DD*/*S* | *S* | *ID* |  |  | *S* |  |  | *ID* |  | *ID* | *ID* | *ID* |  |  |  |  |  |  |  |  |  |  |  |  |
| Amino acids | E | V | F | M | T | S | V | I | G | I | V | V | L | S | L | S | Q | E | E | T |  |  |  |  |  |  |  |  |  |  |  |
|  | D | A | Y | L | P | A | L | L | A | M | I | A | I | T | I | C | E | D | D | A |  |  |  |  |  |  |  |  |  |  |  |
|  |  | L |  |  |  |  |  |  |  |  |  |  |  |  |  | G |  |  |  | E |  |  |  |  |  |  |  |  |  |  |  |
|  |  | I |  |  |  |  |  |  |  |  |  |  |  |  |  | T |  |  |  |  |  |  |  |  |  |  |  |  |  |  |  |

[105] Knight S, Andersson I, Brändén CI. Crystallographic analysis of ribulose1,5-bisphosphate carboxylase from spinach at 2·4 Å resolution: Subunit interactions and active site. J Mol Biol. 1990;215:113–160.

[106] Kellogg E, Juliano N. The structure and function of Rubisco and their implications for systematic studies. Am J Bot. 1997;84:413–413.

[107] Duff AP, Andrews TJ, Curmi PM. The transition between the open and closed states of rubisco is triggered by the inter-phosphate distance of the bound bisphosphate. J Mol Biol. 2000;298:903–916.

[108] Portis AR. Rubisco activase–Rubisco's catalytic chaperone. Photosynth Res. 2003;75:11–27.

[109] Li C, Salvucci ME, Portis AR. Two residues of Rubisco activase involved in recognition of the Rubisco substrate. J Biol Chem. 2005;280:24864–24869.
