# Supplementary material for: Exploring molecular evolution of Rubisco in C3 and CAM Orchidaceae and Bromeliaceae
Source: BMC Evol Biol. 2020 Jan 22;20:11. doi: 10.1186/s12862-019-1551-8 (PMC6977233; doi:10.1186/s12862-019-1551-8)

**Additional file 5: Figure S1**. Decision trees (DT) resolved for each Rubisco L-subunit variable site (with xerror < 1) as a function of the external variables leaf δ^13^C (‰) and habitat preference for the orchid species of the database built with R package ‘rpart’ [55]. Numbers above each tree correspond to the Rubisco L-subunit variable site according to the spinach sequence (AJ400848.1). First level presents the proportion of amino acids in each variable site (brackets). The external variable that allows the best separation of species is shown over the line. The second level presents the distribution of amino acids (in brackets) after the first split. Subsequent divisions are performed until the lowest xerror for the entire DT is obtained (symbolized as squares). Taking as an example the Rubisco L-subunit variable site 225, the first level shows the separation of the 79 species between those that present I (31) and those that present L (48). Over the line, δ^13^C is indicated as the external variable that gives the best split among the three external variables, with species with δ^13^C ≥ -16.35 ‰ having a proportion of I/L of 10/2. On the other hand, the species with δ^13^C < -16.35 ‰ present a proportion of I/L of 21/46. The relative importance of each external variable is calculated and shown in Table 3.


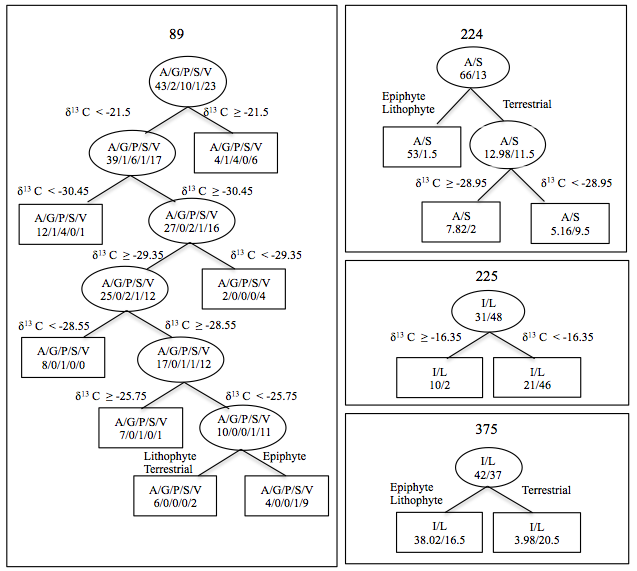

Supplement: Supplementary file 5 — Additional file 5: Figure S1. Decision trees (DT) resolved for each Rubisco L-subunit variable site as a function of the external variables leaf δ13C (‰) and habitat preference for the orchids dataset. [file 12862_2019_1551_MOESM5_ESM.docx]
