# Supplementary material for: Exploring molecular evolution of Rubisco in C3 and CAM Orchidaceae and Bromeliaceae
Source: BMC Evol Biol. 2020 Jan 22;20:11. doi: 10.1186/s12862-019-1551-8 (PMC6977233; doi:10.1186/s12862-019-1551-8)

**Additional file 6: Figure S2**. Decision trees (DT) structure resolved for each variable site (with xerror < 1) as a function of the external variables leaf δ^13^C (‰) and habitat preference based on the bromeliads dataset. See Additional file 5: Figure S1 for detailed explanation on DTs.


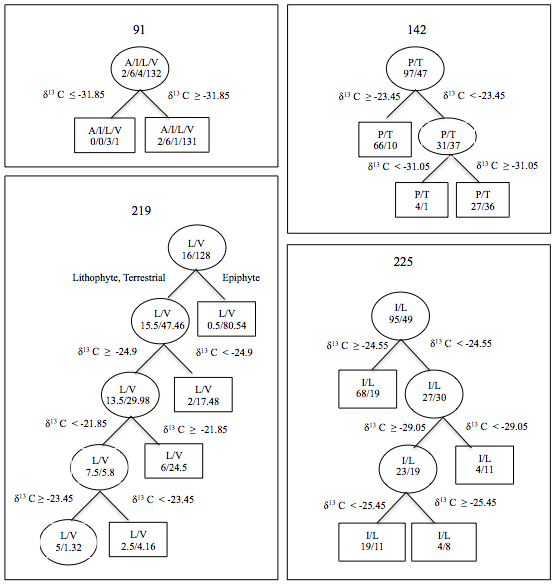


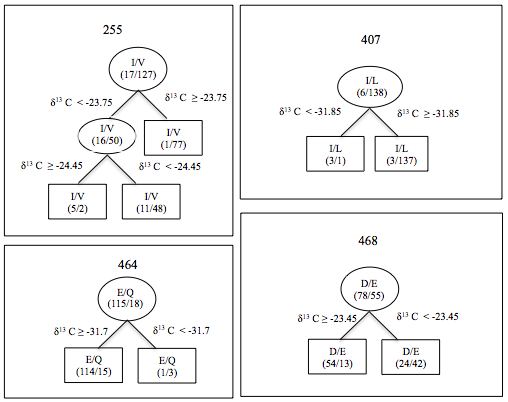

Supplement: Supplementary file 6 — Additional file 6: Figure S2. Decision trees (DT) structure resolved for each variable site as a function of the external variables leaf δ13C (‰) and habitat preference based on the bromeliads dataset. [file 12862_2019_1551_MOESM6_ESM.docx]
